# Novel hybrids of thiazolidinedione-1,3,4-oxadiazole derivatives: synthesis, molecular docking, MD simulations, ADMET study, *in vitro*, and *in vivo* anti-diabetic assessment†

**DOI:** 10.1039/d2ra07247e

**Published:** 2023-01-09

**Authors:** Mahendra Gowdru Srinivasa, Jagdish Gopal Paithankar, Sumit Rao Saheb Birangal, Aravinda Pai, Vasudev Pai, Shridhar N. Deshpande, B. C. Revanasiddappa

**Affiliations:** a Department of Pharmaceutical Chemistry, NGSM Institute of Pharmaceutical Sciences (NGSMIPS), Nitte (Deemed to Be University) Mangalore-575018 Karnataka India revan@nitte.edu.in; b Division of Environmental Health and Toxicology, Nitte University Centre for Science Education and Research (NUCSER), Nitte (Deemed to Be University) Mangalore-575018 Karnataka India; c Department of Pharmaceutical Chemistry, Manipal College of Pharmaceutical Sciences, Manipal Academy of Higher Education (MAHE) Manipal-5761042 Karnataka India; d Department of Pharmacognosy, Manipal College of Pharmaceutical Sciences, Manipal Academy of Higher Education (MAHE) Manipal-5761042 Karnataka India

## Abstract

As compared to standard medicinal compounds, hybrid molecules that contain multiple biologically active functional groups have greater affinity and efficiency. Hence based on this concept, we predicted that a combination of thiazolidinediones and 1,3,4-oxadiazoles may enhance α-amylase and α-glucosidase inhibition activity. A series of novel 3-((5-phenyl-1,3,4-oxadiazol-2-yl)methyl)thiazolidine-2,5-dione derivatives (5a–5j) were synthesized and characterized using different spectroscopic techniques *i.e.*, FTIR, ^1^H-NMR, ^13^C-NMR and MS. To evaluate *in silico*, molecular docking, MMGBSA, and MD simulations were carried out which were further evaluated *via in vitro* inhibition of α-amylase and α-glycosidase enzyme inhibition assays. In addition, the *in vivo* study was performed on a genetic model of *Drosophila melanogaster* to assess the antihyperglycemic effects. The compounds (5a–5j) demonstrated α-amylase and α-glucosidase inhibitory activity in the range of IC_50_ values 18.42 ± 0.21–55.43 ± 0.66 μM and 17.21 ± 0.22–51.28 ± 0.88 μM respectively when compared to standard acarbose. Based on the *in vitro* studies, compounds 5a, 5b, and 5j were found to be potent against both enzymes. *In vivo* studies have shown that compounds 5a, 5b, and 5j lower glucose levels in Drosophila. These compounds could be further developed in the future to produce a new class of antidiabetic agents.

## Abstract 2

As compared to standard medicinal compounds, hybrid molecules that contain multiple biologically active functional groups have greater affinity and efficiency.

## Graphical abstract

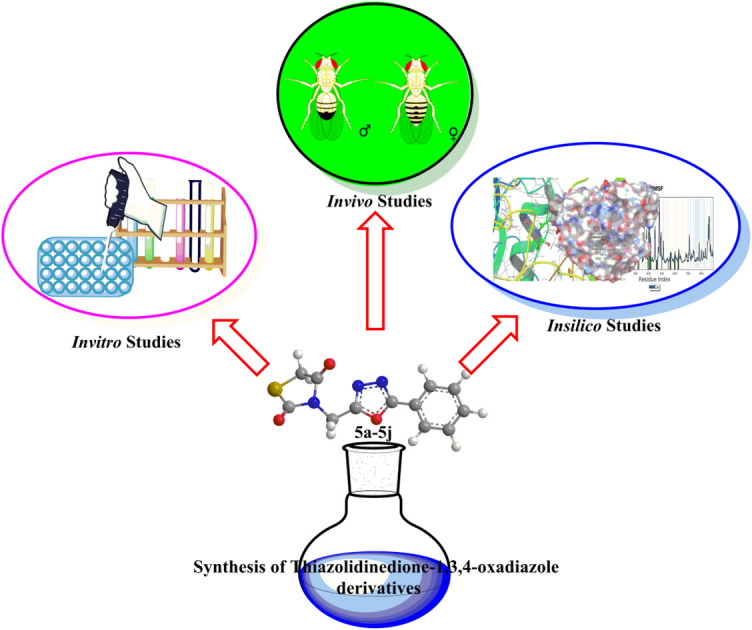

## Introduction

**Fig. 1:**
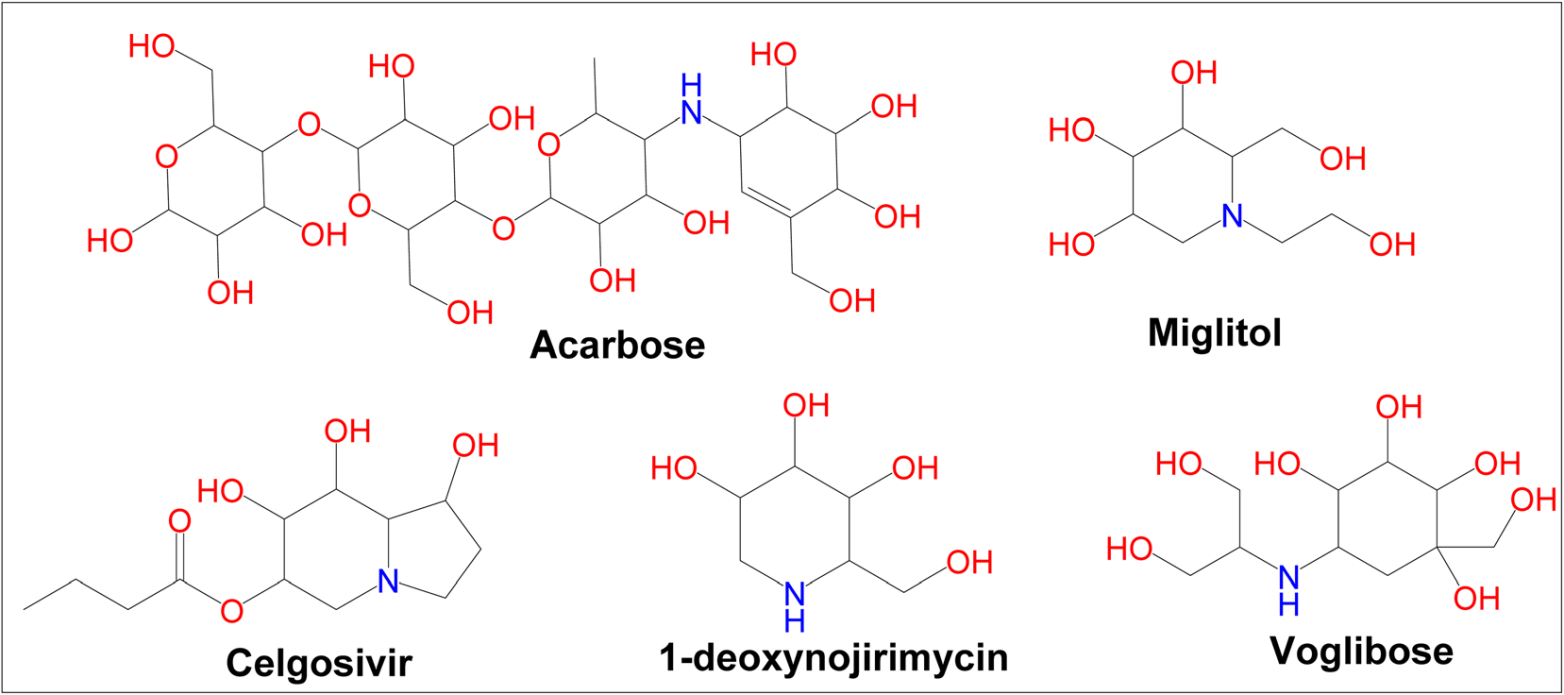
Currently available α-glucosidase and α-amylase inhibitors.

Diabetes mellitus (DM) is a chronic metabolic disorder that affects a large number of people.^1^ In developed countries, the prevalence of diabetes is likely to rise by 20% among adults and 69% in developing countries by 2030.^2^ Glycation of body proteins occurs during diabetes, which results in secondary complications for vital organs. By maintaining ideal blood glucose levels, this process can be inhibited or lowered. Diabetes is treated with insulin and various oral anti-diabetic medications, such as sulphonylureas (*e.g.*, glibenclamide), glinides (*e.g.*, repaglinide), and biguanides (*e.g.*, metformin), thiazolidinediones (*e.g.*, pioglitazone), and α-amylase, α-glycosidase inhibitors like acarbose, voglibose, miglitol, *etc.* Each class of anti-diabetic drugs exerts its effect differently.^3^ There are also some adverse effects such as hypoglycemia, lactic acidosis, liver trouble, and diarrhea caused by these drugs, and they fail to significantly improve diabetic complications. A new class of compounds must be developed, that are more effective with fewer side effects to overcome these problems.^4^ As part of the present study, digestive enzymes α-amylase and α-glucosidase were selected as the best targets for the treatment.^5,6^ The α-amylase hydrolyzes polysaccharides, such as starch, into glucose and maltose^7^ followed by intestinal α-glucosidase enzymes to absorb it into our body.^8^ The blood glucose level is modulated by α-amylase inhibitors after a meal. Hence, potential inhibitors of an α-amylase enzyme could be used as chemotherapeutic agents to treat diabetes,^9^ additionally, the use of multiple approaches, to diabetes treatment can include α-glucosidase enzyme inhibitors.^10^ Because these inhibitors block the enzyme, digestion is slowed; this results in slow glucose absorption. Clinically, acarbose and miglitol both lower postprandial blood glucose levels in the body by inhibiting α-glucosidase. The current type-2 diabetes treatment is acarbose, a tetrasaccharide mimic that inhibits the enzymes α-amylase and α-glucosidase.^11^ Moreover, α-amylase and α-glucosidase inhibitors have been found to possess a very large range of biological activities in contrary to antidiabetic drugs such as acarbose, miglitol, celgosivir, 1-deoxynojirimycin, and voglibose (Fig. 1). For example, celgosivir displays antiviral activity against hepatitis C & B viruses, and 1-deoxynojirimycin exhibits anticancer activity.^12^

The biological significance of α-amylase and α-glucosidase, along with the inefficiency of currently available drugs make the development of novel inhibitors interesting and challenging.^13^ Medicinal chemists are particularly interested in heterocyclic compounds because of their chemical and biological versatility. Therefore, we explored the potential pharmacological effects of heterocyclic organic molecules of various classes and categories as part of our research activity. Thiazolidinedione (TZD) is an attractive pharmacophore to medicinal chemists and pharmaceutical chemists. TZD and its derivatives have a wide range of pharmacological activities, including anti-alzheimer,^14^ anti-bacterial,^15^ anti-fungal,^16^ antiviral,^17^ anti-cancer,^18^ immunosuppressive,^19^ antidiabetic^20^, *etc.*

In addition to being an important drug discovery strategy, 1,3,4-oxadiazoles, have a significant impact on numerous findings, such as anti-diabetic,^21^ anti-obesity,^22^ and anti-cancer.^23^ In light of the therapeutic importance of TZD and 1,3,4-oxadiazole moieties, the present study describes the synthesis of a new class of heterocyclic molecules in which both of the molecules are present, as well as an attempt, was made to develop potential bioactive counterparts. The design process involves the use of a new concept. In this study, two active pharmacophores are coupled to create a new structure that exhibits greater activity than any of the previously used pharmacophores. To enhance its potency, we have clubbed thiazolidinedione with 1,3,4-oxadiazole rings with CH_2_-linker. The addition of methylene linker increases biological activity. The biochemical actions of these newly synthesized molecules were evaluated for their role in mediating inhibition of α-amylase and α-glucosidase activities using the *in silico*, *in vitro* and *in vivo* studies and also by MD simulation study.

## Experimental

### Chemistry

Reagents and chemicals were purchased from Sigma Aldrich, Himedia, and SD Fine Chemicals, Mumbai India. Thin-layer chromatography (TLC) was used to examine the homogeneity of the compounds using *n*-hexane and ethyl acetate as a solvent system (2 : 3). The spots were visualized in the UV chamber using pre-coated silica GF_254_ plates. Equiptronics digital melting point apparatus was used for determining the melting points, and the results are uncorrected. FT-IR spectrum (cm^−1^) was recorded with an Alpha Bruker IR spectrometer. ^1^H-NMR spectra were recorded using JeolECZ 400 FT-NMR spectrometer operating at 400 MHz and ^13^C-NMR spectra were recorded using JeolECZ 400 FT-NMR spectrometer operating at 100 MHz. Trimethylsilyl (TMS) was used (*δ* ppm) as an internal standard. Mass spectra were recorded using the Shimadzu LC-MS-8030 series.

### Synthesis of 1,3-thiazolidine-2,4-dione (1)

Chloroacetic acid (0.6 mmol) was dissolved in 6 mL of distilled water and thiourea (0.6 mmol) was dissolved in 6 mL of distilled water and was mixed. The reaction mixture was stirred for 15 minutes at 400 rpm. A white precipitate appeared on cooling in an ice bath. The mixture was heated with stirring (400 rpm) for 10 hours, after adding 6 mL of concentrated hydrochloric acid. The precipitated white compound was filtered, washed, and recrystallized with alcohol.^24^

### Synthesis of the potassium salt of 1,3-thiazolidine-2,4-dione (2)

A solution of 1,3-thiazolidine-2,4-dione (1) (0.157 mmol) in ethanol (3.0 mL) was mixed with a solution of potassium hydroxide (0.173 mmol) in ethanol (2.3 mL). An ice bath was used to cool the mixture after stirring (300 rpm) for 2 hours. The crystalline solid was filtered, washed with ethanol, dried, and recrystallized from alcohol.^24^

### Synthesis of ethyl 2,4-dioxo-1,3-thiazolidin-3-yl acetate (3)

The potassium salt of 1,3-thiazolidine-2,4-dione (2) (0.066 mmol) was dissolved in dimethylformamide (30 mL) and slowly, ethyl chloroacetate (0.066 M, 7.06 mL) was added. The reaction mixture was refluxed by stirring at 400 rpm for 10 hours. After pouring the mixture into 200 mL of ice-cold water, a reddish-orange liquid gets separated. This was added to 20 mL chloroform, washed with distilled water (three times), and desiccated in anhydrous sodium sulphate. The residual oil was distilled out from the solvent which was extracted under low pressure.

### Synthesis of 2-2,4-dioxo-1,3-thiazolidin-3-yl acetohydrazide (4)

The ethyl 2,4-dioxo-1,3-thiazolidin-3-yl acetate (3) was added to absolute ethanol (10 mL), followed by the addition of hydrazine hydrate (0.082 mmol, 99% w/w, 4 mL). The mixture was refluxed for 6 hours and cooled to room temperature. The precipitate was filtered, dried, and re-crystallized using ethanol. (Light brown solid) MP: 192–194 °C; IR (KBr) *ν*_max_: cm^−1^; ^1^H-NMR (400 MHz, DMSO): (ppm): 9.25 (s, NH, 1H), 4.47 (s, CH_2_, 2H), 4.23 (s, NH_2_, 2H), 4.14 (s, CH_2_, 2H): ^13^C NMR (DMSO-d_6_, 100 MHz) *δ* (ppm); 171.0, 168.6, 162.4, 52.4, and 34.6. LC-MS (*m*/*z*): calculated for C_5_H_8_N_3_O_3_S is 189.1; found: 189.1 (M+).

### Synthesis of 3,5-phenyl-1,3,4-oxadiazol-2-yl-methyl thiazolidine-2,5-dione (5a–5j)

To 1 mmol acetohydrazide (4) (5 mL phosphorous oxychloride) with 1 mmol of aromatic acids (4) were added and the mixture was refluxed for about 12–16 hours. After cooling to room temperature, it was poured into crushed ice and the precipitated compound was filtered and neutralized with 20% NaHCO_3_ the resulting solid mass was filtered, washed with water, and recrystallized in alcohol.^25^

#### 3-((5-(2,4-Dichlorophenyl)-1,3,4-oxadiazol-2-yl)methyl)thiazolidine-2,4-dione (5a)

Molecular formula: C_12_H_7_Cl_2_N_3_O_3_S, yellow solid, yield: 78%, melting point: 210–212 °C; FT-IR (cm^−1^): 3020 (Ar, C–H), 2927 (CH_2_), 1734 (C=O), 1585 (C=N), 1513 (C=C). ^1^H-NMR (400 MHz, DMSO-d_6_, *δ*, ppm): 8.78–7.10 (m, Ar–H, 3H), 4.33 (s, CH_2_, 2H), 3.26 (s, CH_2_, 2H): ^13^CNMR (100 MHz, DMSO-d_6_, *δ*, ppm): 165.3, 164.2, 163.4, 162.2, 135.0, 129.2, 128.6, 123.3, 122.6, 119.7, 46.3, and 39.0: LC-MS (*m*/*z*): calculated for C_12_H_7_Cl_2_N_3_O_3_S is 344.1; found: 344.1 (M+).

#### 3-((5-(4-Bromophenyl)-1,3,4-oxadiazol-2-yl)methyl)thiazolidine-2,4-dione (5b)

Molecular formula: C_12_H_8_BrN_3_O_3_S, yellow solid, yield: 87%, melting point: 185–187 °C; FT-IR (cm^−1^): 3003 (Ar, CH), 2924 (CH_2_), 1735 (C=O), 1586 (C=N), 1516 (C=C), 742 (C–Br). ^1^H-NMR (400 MHz, DMSO-d_6_, *δ*, ppm): 7.69–7.11 (m, Ar–H, 4H), 4.41 (s, CH_2_, 2H), 3.36 (s, CH_2_, 2H): ^13^CNMR (100 MHz, DMSO-d_6_, *δ*, ppm): 165.9, 159.9, 156.9, 149.8, 129.3, 128.7, 128.6, 123.9, 123.4, 119.7, 45.8, and 34.5: LC-MS (*m*/*z*) calculated for C_12_H_8_BrN_3_O_3_S is 354.1; found: 354.1 (M+).

#### 3-((5-(4-Aminophenyl)-1,3,4-oxadiazol-2-yl)methyl)thiazolidine-2,4-dioneone (5c)

Molecular formula: C_12_H_10_N_4_O_3_S, yellow solid, yield: 82%, melting point: 198–200 °C; FT-IR (cm^−1^): 3336 (–NH), 3007 (Ar, CH), 2932 (CH_2_), 1712 (C=O), 1591 (C=N), 1512 (C=C). ^1^H-NMR (400 MHz, DMSO-d_6_, *δ*, ppm): 8.78–8.14 (m, Ar–H, 4H), 7.10 (s, NH_2,_ 2H), 4.98 (s, CH_2_, 2H), 3.36 (s, CH_2_, 2H): ^13^CNMR (100 MHz, DMSO-d_6_, *δ*, ppm): 166.0, 164.6, 163.3, 161.2, 144.8, 135.0, 129.2, 128.6, 123.3, 119.4, 45.4, and 33.6: LC-MS (*m*/*z*): calculated for C_12_H_10_N_4_O_3_S is 290.2; found: 290.2 (M+).

#### 3-((5-(4-Hydroxyphenyl)-1,3,4-oxadiazol-2-yl)methyl)thiazolidine-2,4-dione (5d)

Molecular formula: C_12_H_9_N_3_O_4_S, yellow solid, yield: 73%, melting point: 192–194 °C; FT-IR (cm^−1^): 3315 (–OH), 3020 (Ar, C–H), 2927 (CH_2_), 1734 (C=O), 1585 (C=N), 1513 (C=C). ^1^H-NMR (400 MHz, DMSO-d_6_, *δ*, ppm): 9.41 (s, OH, 1H), 8.19–7.11 (m, Ar–H, 4H), 4.54 (s, CH_2_, 2H), 3.35 (s, CH_2_, 2H): ^13^CNMR (100 MHz, DMSO-d_6_, *δ*, ppm): 165.4, 164.6, 163.4, 161.7, 149.8, 135.0, 129.3, 128.6, 123.4, 119.7, 45.4, and 34.6: LC-MS (*m*/*z*): calculated for C_12_H_9_N_3_O_4_S is 291.2; found: 291.2 (M+).

#### 3-((5-Phenyl-1,3,4-oxadiazol-2-yl)methyl)thiazolidine-2,4-dione (5e)

Molecular formula: C_12_H_9_N_3_O_3_S, yellow solid, yield: 69%, melting point: 215–217 °C; FT-IR (cm^−1^): 3017 (Ar, CH), 2857 (CH_2_), 1736 (C=O), 1598 (C=N), 1515 (C=C). ^1^H-NMR (400 MHz, DMSO-d_6_, *δ*, ppm): 8.40–7.11 (m, Ar–H, 5H), 3.35 (s, CH_2_, 2H), 3.05 (s, CH_2_, 2H): ^13^CNMR (100 MHz, DMSO-d_6_, *δ*, ppm): 167.0, 165.7, 163.2, 162.1, 131.5, 129.3, 128.6, 124.4, 123.3, 119.7, 45.8, and 35.2: LC-MS (*m*/*z*): calculated for C_12_H_9_N_3_O_3_S is 275.2; found: 275.2 (M+).

#### 3-((5-(4-Methoxyphenyl)-1,3,4-oxadiazol-2-yl)methyl)thiazolidine-2,4-dione (5f)

Molecular formula: C_13_H_11_N_3_O_4_S, yellow solid, yield: 86%, melting point: 178–180 °C; FT-IR (cm^−1^): 3046 (Ar, CH), 2896 (CH_2_), 1687 (C=O), 1584 (C=N), 1515 (C=C). ^1^H-NMR (400 MHz, DMSO-d_6_, *δ*, ppm): 8.16–7.07 (m, Ar–H, 4H), 4.34 (s, CH_2_, 2H), 3.83 (s, OCH_3,_ 3H), 3.32 (s, CH_2_, 2H): ^13^CNMR (100 MHz, DMSO-d_6_, *δ*, ppm): 168.8, 166.2, 165.1, 163.0, 161.8, 128.5, 124.0, 123.3, 119.7, 53.4, 45.4, and 33.6: LC-MS (*m*/*z*): calculated for C_13_H_11_N_3_O_4_S is 305.3; found: 305.3 (M+).

#### 3-((5-(4-Chlorophenyl)-1,3,4-oxadiazol-2-yl)methyl)thiazolidine-2,4-dione (5g)

Molecular formula: C_12_H_8_ClN_3_O_3_S, yellow solid, yield: 81%, melting point: 172–174 °C; FT-IR (cm^−1^): 3035 (Ar, CH), 2920 (CH_2_), 1731 (C=O), 1592 (C=N), 1517 (C=C). ^1^H-NMR (400 MHz, DMSO-d_6_, *δ*, ppm): 8.16–7.09 (m, Ar–H, 4H), 3.89 (s, CH_2_, 2H), 3.38 (s, CH_2_, 2H): ^13^CNMR (100 MHz, DMSO-d_6_, *δ*, ppm): 167.9, 164.7, 162.2, 161.7, 142.3, 135.0, 129.2, 128.6, 123.3, 119.7, 46.7, and 34.4: LC-MS (*m*/*z*): calculated for C_12_H_8_ClN_3_O_3_S is 309.7; found: 309.3 (M+).

#### 3-((5-(2-Chloro-4-nitrophenyl)-1,3,4-oxadiazol-2-yl)methyl)thiazolidine-2,4-dione (5h)

Molecular formula: C_12_H_7_ClN_4_O_5_S, yellow solid, yield: 74%, melting point: 161–163 °C; FT-IR (cm^−1^): 3046 (Ar, CH), 2924 (CH_2_), 1734 (C=O), 1585 (C=N), 1514 (C=C). ^1^H-NMR (400 MHz, DMSO-d_6_, *δ*, ppm): 8.19–7.11 (m, Ar–H, 3H), 4.14 (s, CH_2_, 2H), 3.36 (s, CH_2_, 2H): ^13^CNMR (100 MHz, DMSO-d_6_, *δ*, ppm): 165.9, 164.3, 161.9, 161.0, 149.8, 135.0, 129.3, 128.6, 123.4, 119.7, 44.8, and 34.5: LC-MS (*m*/*z*): calculated for C_12_H_7_ClN_4_O_5_S is 354.7; found: 354.7 (M+).

#### 3-((5-(4-Nitrophenyl)-1,3,4-oxadiazol-2-yl)methyl)thiazolidine-2,4-dione (5i)

Molecular formula: C_12_H_8_N_4_O_5_S, yellow solid, yield: 81%, melting point: 153–155 °C; FT-IR (cm^−1^): 3005 (Ar, CH), 2955 (CH_2_), 1735 (C=O), 1582 (C=N), 1514 (C=C). ^1^H-NMR (400 MHz, DMSO-d_6_, *δ*, ppm): 8.12–7.69 (m, Ar–H, 4H), 3.83 (s, CH_2_, 2H), 3.02 (s, CH_2_, 2H): ^13^CNMR (100 MHz, DMSO-d_6_, *δ*, ppm): 164.9, 164.0, 163.2, 162.1, 149.2, 135.105, 129.3, 128.6, 123.4, 119.7, 44.7, and 34.4: LC-MS (*m*/*z*): calculated for C_12_H_8_N_4_O_5_S is 320.2795; found: 320.2735 (M+).

#### 3-((5-(2,4-Dihydroxyphenyl)-1,3,4-oxadiazol-2-yl)methyl)thiazolidine-2,4-dione (5j)

Molecular formula: C_12_H_9_N_3_O_5_S, yellow solid, yield: 73%, melting point: 204–206 °C; FT-IR (cm^−1^): 3326 (–OH), 3005 (Ar, CH), 2955 (CH_2_), 1769 (C=O), 1591 (C=N), 1521 (C=C). ^1^H-NMR (400 MHz, DMSO-d_6_, *δ*, ppm): 10.14 (s, OH, 2H), 7.92–7.09 (m, Ar–H, 4H), 4.43 (s, CH_2_, 2H), 3.32 (s, CH_2_, 2H): ^13^CNMR (100 MHz, DMSO-d_6_, *δ*, ppm): 165.5, 164.6, 161.4, 149.3, 135.1, 130.4, 128.3, 122.9, 119.6, 45.4, and 34.5: LC-MS (*m*/*z*): calculated for C_12_H_9_N_3_O_5_S is 307.2; found: 307.2 (M+).

### *In silico* analysis

#### Docking studies

The docking study was performed using the Glide module from Schrodinger 2020-3 suite Maestro (https://www.schrodinger.com) device installed on a Linux workstation.

#### Ligand preparation

Chemdraw database (http://www.cambridgesoft.com) was used to obtain the designed compounds, which were arranged using the Schrodinger suite's LigPrep module (https://www.schrodinger.com). To ensure consistent biological activity, the high-energy ionization and tautomers were excluded during the production process with the Epik tool. We simulated the ligands before using virtual screening, to identify and separate the ligands that did not obey Lipinski's rules by using the Qikprop module (https://www.schrodinger.com/products/qikprop).

#### Preparation of protein

The protein was prepared using the preparation wizard tool. The structure of the glucoamylase enzyme in 3D form was obtained from the RCSB Protein Data Bank (https://www.rcsb.org). The PDB ID: 3TOP shows glucoamylase at a resolution of 2.3 Å treated by adding missing hydrogen, assigning regular bonding format, treating for metal; removing molecules of water which lie 5 Å apart from their heterogeneous groups. The bonds of hydrogen were optimized using sample orientation. All polar group hydrogen was displayed and the structure of the protein was finally minimized to its default root mean square deviation value of 0.30 Å.^26^

#### Protein–ligand docking

The receptor grid denotes the region of the target protein, where the ligand sampling was performed during the process of molecular docking. It was established using the receptor grid generation interface in the Glide tool of Maestro. It was performed using the force field of OPLS-2005. Using LigPrep defined receptor folders from the grid were selected and docking was made flexible with the extra precision (XP) option on the Glide module. These bindings were ranked on computerized scores consisting of the grid score, Glide score (proprietary), and energy strain (internal). The results were viewed on the pose-viewer in the form of structural output formats. For predicting the binding affinity and ligand ranking, the Glide score was used. The molecular docking study was conducted on the extra precision (XP) mode of the docking program of α-amylase and α-glucosidase inhibitors.

#### Molecular mechanics/generalized Born surface area (MM/GBSA) assay

The Glide docking was performed in XP mode without applying any constraints. A local optimization feature minimizes the docked poses (Prime v4.9). The MM-GBSA continuum solvent model computes the binding free energies of the docked complexes. This incorporates the OPLS3 force field, a VSGB 2.0 implicit solvent model, and the rotamer search algorithms.^27^

#### Molecular dynamics (MD) simulation studies

Molecular dynamics simulations were performed using the Schrödinger LLC package (https://www.schrodinger.com/products/desmond)^28^ and the studies were carried out for 100 ns on compound 5j based on Glide dock XP scores using the Maestro 12.6 Desmond panel. 1000 frames were produced and intervals of 20 ps were reported during these simulation studies. To gain a better understanding of ligand–protein interactions and their binding affinities, dynamic experiments were used. Under orthorhombic boundary conditions, systems for the 3TOP–5j complexes were designed using a predefined SPC solvation model. The buffering approach was used to measure the box size. To neutralize the charges on the models, sodium and chloride ions were used. The designed systems were subjected to energy minimization using 2000 iterations and a convergence threshold of 25 kcal mol^−1^ Å^−1^. NPT ensemble class simulations of the minimized complexes were carried out at a 300 K temperature and 1 bar pressure.^29^

#### Evaluation of ADME-toxicity of compounds (5a–5j)

The ADMET properties of all the new compounds were predicted by using the Qikprop module, to find out the drug-like properties. QikProp module version 5.4 of Maestro, Schrodinger (https://www.schrodinger.com/products/qikprop) was used to calculate the molecular descriptor and to predict the ADME profile of these compounds.

#### Drug-likeness prediction

The OSIRIS property explorer (https://www.organic-chemistry.org/prog/peo) generally uses chemical structures and then calculates various drug-relevant properties on valid structures. The resulting predictions are given values and color codes. The TPSA, log *S* calculation, *c* log *P* calculation, molecular weight, drug score, fragment based drug-likeness, and toxicity are some of the properties analyzed.^30^

### Biological activity

#### *In vitro* α-amylase inhibitory assay

The reaction mixture contains 20 μL of α-amylase solution (0.5 mg mL^−1^) and 200 μL of sodium phosphate buffer (pH 6.9, 0.02 M). Test samples (10–50 g mL^−1^) were added (250 μL) to the reaction mixture following which; incubation was carried out for 10 min at room temperature. This was accompanied by the addition of 200 μL of 1% starch solution, and further incubated for 10 min at 25 °C. By adding 400 μL of dinitrosalicylic acid (DNS) reagent, the reaction was terminated. Lastly, it was incubated in water (70 °C, 5 min). The absorbance was recorded using an ELISA microplate reader at 540 nm. As a standard, acarbose was used. All the experiments were performed in triplicates. It was the reaction mixture without the test sample that served as the control for the test.^31^

The percent inhibition was calculated as:% Inhibition = [(*C* − *T*)/*C*] × 100*C* – absorbance of control, *T* – absorbance of sample.

#### *In vitro* α-glycosidase inhibitory assay

The reaction mixture contains 50 μL of 0.1 M phosphate buffer (pH = 6.8), 10 μL of α-glucosidase enzyme and 20 μL of test samples (10–50 μg mL^−1^) were taken in a 96-well plate and incubated at 37 °C for 15 min. Furthermore, 20 μL of *p*-nitrophenyl d-glucopyranoside solution was added as a substrate and incubated for an additional 20 min at 37 °C. 50 μL of 0.1 M Na_2_CO_3_ is added to terminate the reaction. The liberated *p*-nitrophenol was measured at 405 nm with an Elisa microplate reader. Acarbose served as the standard and control was set up in parallel without the test substance. All the experiments were performed in triplicates.^31^

The inhibition was calculated in percentage as:% Inhibition = [(*C* − *T*)/*C*] × 100*C* – absorbance of control, *T* – absorbance of sample.

#### Drosophila culture and experimental design

In this study, Oregon-R flies were obtained directly from the Nitte University, Drosophila Lab, Mangalore, India, and maintained on a wheat cream-agar medium with a relative humidity of 65–70%, and a light–dark cycle of twelve hours each. Separation of males and females of one-day-old flies were cultured in, food containing high sugar diet (HSD), and different groups of food containing HSD with uniform concentrations (18 μg g^−1^ feed) of the compounds 5a, 5b, 5j for 10 days. Acarbose was administered as a positive control. Twenty flies were taken and they were homogenized in 0.2 mL of ice-cold phosphate buffer saline. (pH 7.4). Homogenized samples were centrifuged at 10 000*g* for 15 min at 4 °C. All fly homogenates were tested for glucose concentration using the manufacturer's protocol (Agape Diagnostics Ltd, India). Throughout the experiments, the control group was maintained in the same conditions as that of other groups except for HSD and compounds. All experiments were carried out in triplicates and the results were expressed in percentages respective to the control group.^32^

#### Data analysis

The results of all the experiments (*n* = 3) were analyzed and expressed as mean ± standard deviation (SD). The Student's *t*-test was performed using GraphPad Prism software (GraphPad Software Inc., CA, USA). *p* values ≤ 0.05 were considered as statistically significant (*p ≤ 0.05; *p ≤ 0.01; **p ≤ 0.001).

#### MTT assay

The cytotoxic concentration of synthetic compounds (5a–5j) was evaluated by MTT assay. A standard MTT assay was performed with various concentrations of synthesized compounds against NIH/3T3 cells using a 96-well microplate reader at 570 nm. The cell lines were cultured in DMEM, supplemented with 10% FBS in flasks, and incubated at 37 °C with 5% CO_2_ in DMEM medium with streptomycin and penicillin (100 IU mL^−1^) at the appropriate concentrations. The 96-well plates with media were incubated. After overnight incubation, each plate was discarded and fresh medium was added to each well with varying concentrations (5 μM, 10 μM, 25 μM, 50 μM, 100 μM) of test samples. Then, 200 μL of MTT (0.5 mg mL^−1^) were put in each well and further incubated for four hours. Afterward, DMSO (100 μL) was transferred into each microplate well. The absorbance was measured at 570 nm to determine how much MTT was reduced to formazan in the cells. The IC_50_ value was recorded for the samples causing 50% cytotoxicity for all of the cell lines.^33^

## Results and discussion

**Fig. 2:**
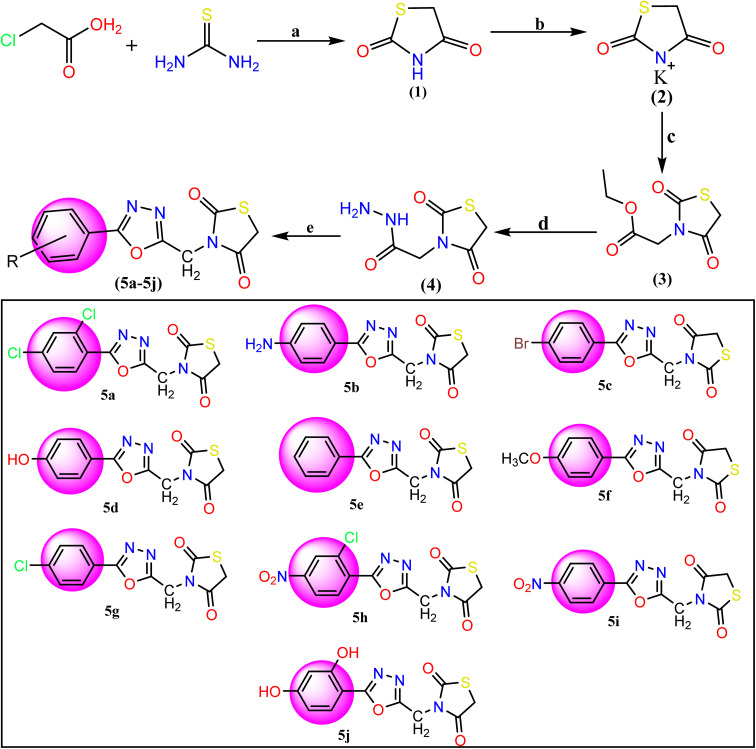
Reagents and conditions for the synthesis of TZD-1,3,4-oxadiazole derivatives (5a–5j): (a) dilute HCl and refluxed at 400 rpm for 10 hours with stirring. (b) Ethanolic KOH at room temperature with stirring, (c) ethyl chloroacetate and DMF, (d) hydrazine hydrate and ethanol mixture were refluxed for 6 h. (e) Aromatic acids and POCl_3_ were added and the mixture was refluxed for about 12–16 h 5a: 2,4-(Cl)_2_, 5b: 4-NH_2_, 5c: 4-Br, 5d: 4-OH, 5e: C_6_H_5_, 5f: 4-OCH_3_, 5g: 4-Cl, 5h: 2-Cl-4-NO_2_, 5i: 4-NO_2_, 5j: 2,4-(OH)_2_.

The synthesis of the titled compounds (5a–5j) has been represented in Fig. 2. All the new compounds and intermediates were purified by successive recrystallization from ethanol. The purity of the compound was confirmed through melting points and TLC using silica gel G plates as stationary phase and *n*-hexane and ethyl acetate as a solvent system (2 : 3). Table 1 lists the physicochemical features of the final synthesized compounds. Based on spectral data, FT-IR, ^1^H NMR, ^13^C-NMR, and LCMS, the possible structures of all the compounds were established.

**Table 1:** Physicochemical properties of compounds (5a–5j)

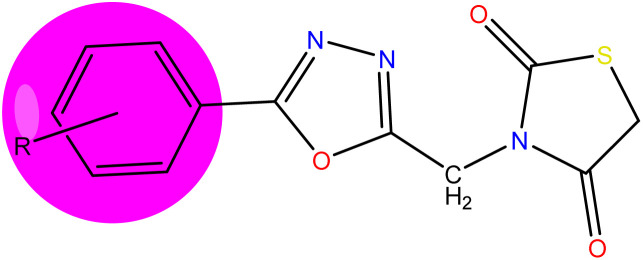					
Compounds	R	Molecular formula	Molecular weight	MP (°C)	Yield (%)
5a	2-4-(Cl)_2_	C_12_H_7_Cl_2_N_3_O_3_S	344.172	210–212	78
5b	4-Br	C_12_H_8_BrN_3_O_3_S	354.177	185–187	87
5c	4-NH_2_	C_12_H_10_N_4_O_3_S	290.296	198–200	82
5d	4-OH	C_12_H_9_N_3_O_4_S	291.281	192–194	73
5e	C_6_H_5_	C_12_H_9_N_3_O_3_S	275.282	215–217	69
5f	4-OCH_3_	C_13_H_11_N_3_O_4_S	305.308	178–180	86
5g	4-Cl	C_12_H_8_ClN_3_O_3_S	309.726	172–174	81
5h	2-Cl-4-NO_2_	C_12_H_7_ClN_4_O_5_S	354.724	161–163	74
5i	4-NO_2_	C_12_H_8_N_4_O_5_S	320.279	153–155	81
5j	2,4-(OH)_2_	C_12_H_9_N_3_O_5_S	307.284	204–206	73

The IR spectra of compound 5a depicted the absorption bands at 3020 for aromatic (C–H), 2927 (CH_2_), and 1611 (C=N), respectively. The presence of C=O absorption bands was observed at 1734 cm^−1^, the key functional group in the thiazolidinedione ring. In the ^1^H-NMR spectra of the compound, 5a revealed the singlet signal for CH_2_ at *δ* 3.26 & 4.33 regions respectively. The aromatic protons were observed as multiplets in the region *δ* 7.10–8.78. The mass spectrum of compound 5a showed a molecular ion peak at *m*/*z* = 344.17 (M+), which is in agreement with the assigned molecular formula C_12_H_7_Cl_2_N_3_O_3_S. The ^13^C-NMR spectra of compound, 5a showed the presence of required carbon atoms and the presence of key lactone groups.

### Docking results

**Fig. 3:**
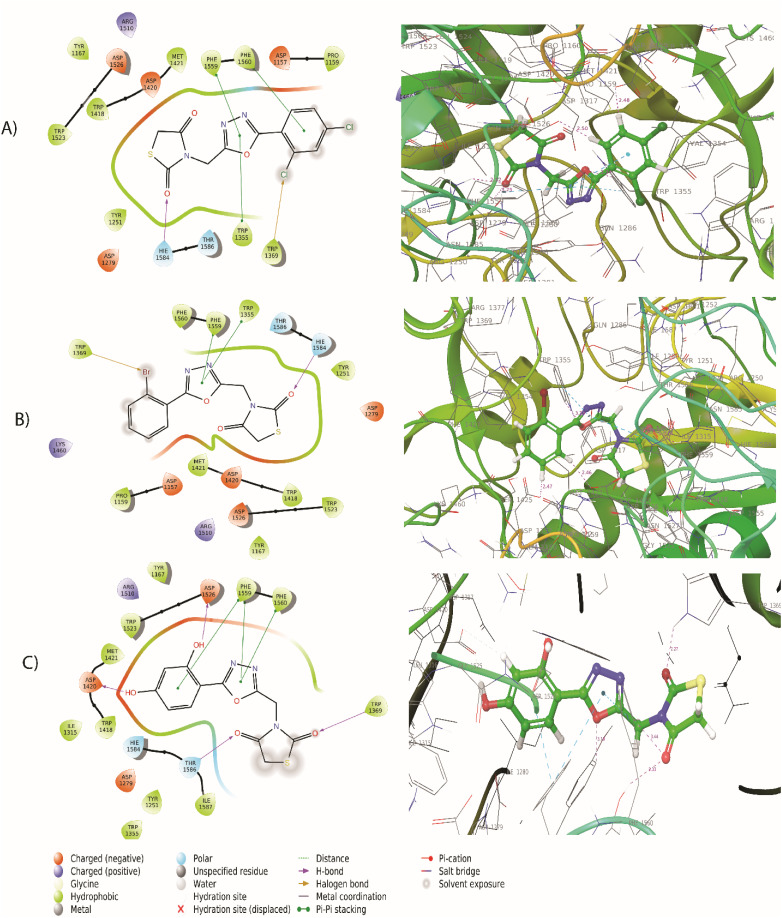
(A) 2D and 3D interaction of compound 5a with 3TOP (B) 2D and 3D interaction of compound 5b with 3TOP (C) 2D and 3D interaction of compound 5j with 3TOP.

The synthesized compounds bind at the active site of the target PDB: 3TOP (the crystal structure of the α-glucoamylase), in the groove of the target binding site. This might be important in understanding their role as a signal transducer, in diabetes which targets α-glucosidase and α-amylase enzymes. We expressed the binding affinity of these derivatives as G scores (Table 2). The binding free energy of all derivatives ranged from −9.942 to −5.085. In terms of binding energies, compounds 5a, 5c, and 5j have the greatest binding scores, with binding energy values of −6.217, −6.039, and −6.56, respectively (Table 2). Acarbose showed the highest binding affinity towards the target with binding free energies of −9.942. The compound 5j which was the most active compound in the dataset presents a conventional hydrogen bond with the carbonyl group in the thiazolidinedione ring with *Thr1369* and *Thr1586* residue. The hydroxyl group of the aromatic ring interacted with *Asp1420*, *Arg1510*, and *Asp1526* residue. The aromatic ring and 1,3,4-oxadiazole ring provides pi–pi linkage interaction with *Phe 1559* and *Phe1560* residue. Other amino acid interactions like *Tyr1251*, *Asp1279*, *Ile1315*, *Trp1355*, *Trp1418*, *Met1471*, *Trp1523*, and *Hie1584* showed in Fig. 3. Compound 5a showed a hydrogen bond with the carbonyl group in the thiazolidinedione ring with *Hie1584* residue. The chlorine group substituted with the aromatic ring of the halogen bond provides interaction with *Trp1569* residue. The aromatic ring and 1,3,4-oxadiazole ring provides pi–pi linkage interaction with *Trp1355*, *Phe1559*, and *Phe1560* residue. Other amino acid interaction includes *Pro1159*, *Asp1157*, *Tyr1167*, *Tyr1251*, *Asp1279*, *Trp1418*, *Asp1420*, *Met1421*, *Lys1460*, *Arg1510*, *Trp1523*, *Asp1526*, and *Thr1526*. The compound 5b had a hydrogen bond with the carbonyl group in the thiazolidinedione ring with *Hie1584* residue. The bromine group substituted with an aromatic ring of halogen bond provided interaction with Trp1369 residue (Fig. 3). The 1,3,4-oxadiazole ring showed pi–pi linkage interaction with *Trp1355* residue. Other amino acid interactions like *Asp1157*, *Pro1159*, *Tyr1251*, *Asp1279*, *Trp1418*, *Asp1420*, *Met1421*, *Lys1460*, *Asp1526*, and *Thr1586*. Based on the docking scores, the selected candidates were found to be good for diabetic activity.

**Table 2:** Glide docking scores (kcal mol^−1^) of the compounds (5a–5j) on enzyme PDB ID: 3TOP

Compounds	Extra-precision docking score (kcal mol^−1^)			
	Gscore^a^	Gemodel^b^	Gecou^c^	evdW^d^
5a	**−6.217**	−61.651	−5.886	−38.494
5b	**−6.039**	−59.164	−5.219	−37.307
5c	−6.037	−57.182	−6.948	−35.563
5d	−5.958	−55.244	−7.573	−35.379
5e	−5.085	−51.949	−2.969	−35.257
5f	−5.885	−55.499	−4.709	−35.959
5g	−5.974	−55.115	−4.937	−35.578
5h	−5.928	−65.157	−6.622	−40.067
5i	−6.026	−57.822	−5.71	−37.578
5j	**−6.56**	−57.559	−13.566	−28.709
Acarbose	−9.942	−87.212	−28.178	−29.573

a Glide score.

b Glideemodel (model energy, emodel).

c Glideecoul (Coulomb energy).

d Glide evdw (van der Waals energy).

### MM/GBSA assay

For the best-ranking molecules, the binding free energy calculations of molecular mechanics/generalized Born surface area (MM/GBSA) were performed. To estimate the relative affinity of ligand binding to the receptor, the Glide XP mode was used. All molecules were with good binding-free energy and their values were observed from −22.39 to −52.32 kcal mol^−1^ (Table 3). To obtain the Δ*G* bind calculations, the optimal poses from XP dock complexes 5a, 5c, and 5j were chosen. The reliability of this approach for grading the affinity of the designed molecules binding to their respective target proteins has been reported. The MM-GBSA score produces a significant correlation with the experimentally determined results. The compounds 5a, 5c, and 5h showed −51.06 kcal mol^−1^, −50.12 kcal mol^−1^, and −52.32 kcal mol^−1^ respectively which are favorable binding energies. However, in the present case, 5h has shown the best binding free energy and the results of the same are shown in Table 3.

**Table 3:** MM-GBSA approach for compounds (5a–5j)^a^

Compounds	Δ*G* bind (kcal mol^−1^)	Δ*G* bind Coulomb	Δ*G* bind covalent	Δ*G* bind van der	Δ*G* bind H bond	Δ*G* bind lipophilic
5a	−51.06	−13.7	3.23	−42.37	−0.35	−27.15
5b	−50.12	−12.15	2.48	−39.79	−0.39	−24.66
5c	−22.39	−16.23	2.47	−37.11	−4.61	−18.22
5d	−22.79	−15.5	2.56	−38.05	−1.49	−18.59
5e	−23.76	−7.49	1.78	−40.35	−0.13	−22.24
5f	−45.56	−9.54	2.48	−40.26	−0.41	−22.98
5g	−48.45	−10.83	3.11	−39.54	−0.39	−24.69
5h	−52.32	−7.58	3.97	−44.13	−1.08	−24.63
5i	−49.11	−3.29	2.64	−41.83	−0.6	−21.34
5j	−29.21	−18.46	4.58	−32.32	−2.35	−18.33
Acarbose	−46.21	−119.36	18.97	−33.45	−8.32	−43.55

a Δ*G* bind: free energy of binding; Δ*G* bind Coulomb: Coulomb energy; Δ*G* bind covalent: covalent energy (internal energy); Δ*G* bind van der: van der Waals energy; Δ*G* bind H bond: hydrogen bonding energy; Δ*G* bind lipophilic: hydrophobic energy (non-polar contribution estimated by solvent accessible surface area).

### Molecular dynamics simulation

**Fig. 4:**
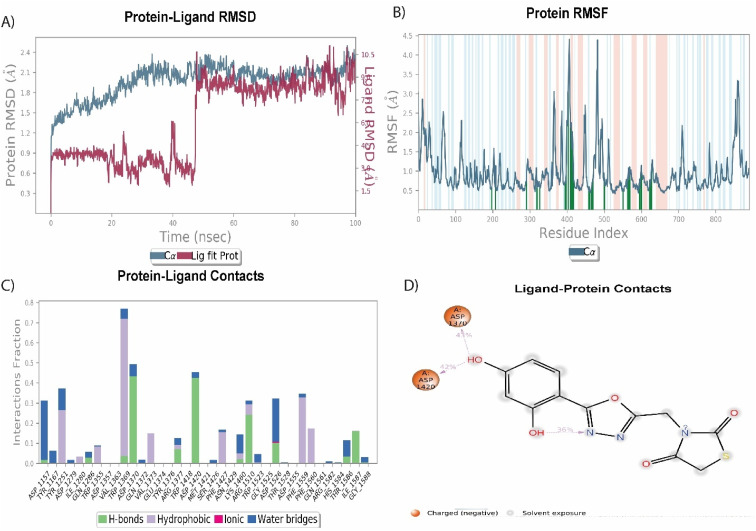
MD simulation analysis of 5j in complex with enzyme active site (PDB ID: 3TOP) (A) RMSD (protein RMSD is shown in grey while RMSD of compound 5j is shown in red), (B) protein RMSF, (C) protein–ligand contact analysis of MD trajectory and (D) 2D interaction diagram.

Among the synthesized molecules, the compound 5jwas further extended for the molecular dynamics simulation study. Based on an explicit hydration environment, a 100 ns MD simulation was performed to assess compound 5j's stability in a complex with 3TOP. MD simulation results were analyzed using root-mean-standard deviation (RMSD), root-mean-square fluctuation (RMSF), and protein–ligand contact mapping. An important parameter of the MD simulation trajectory used to predict Cα deviation in a dynamic environment is protein Cα RMSD. The RMSD of 5j fluctuated in the first phase of the simulation up to 45 ns, then increased to 2.3 Å and equilibrated until the end. A protein's RMSD graph revealed stability from 45 to 100 ns, with an RMSD range of 1.8 to 2.4 Å a good result. Over time, RMSF calculates the average deviation from the initial position for each protein residue. A specific component of the protein structure that deviates from the mean is examined by the RMSF. When protein molecules are bound to a small molecule, the RMSF of each amino acid can be used to determine the stability of the bound protein molecules. When MD simulation is performed, proteins with higher RMSF values indicate greater flexibility, while proteins with lower RMSF values suggest less flexibility. A plot of the RMSF of each amino acid of the 3TOP protein bound to compound 5j is shown in Fig. 4B. The compound 5j contacts 37 amino acids of the 3TOP as shown in Fig. 4B. Protein interactions with the ligand were monitored throughout the simulation. In the stipulated duration, Tyr1251, and Trp1369 demonstrated hydrophobic interaction with compound 5j. Further, hydrogen bond interaction was observed with amino acid residues such as Asp1370 and Asp1420. The MD simulation revealed ionic, amino-acid-mediated hydrogen bond interactions with the compound 5j, the simulation also revealed hydrophobic interaction. Fig. 4C shows the specific sequence contacts between the various amino acid residues present in the complex 5j–3TOP.

### Absorption, distribution, metabolism, and excretion (ADME) properties of compounds (5a–5j) using QikProp

The QikProp pharmacokinetic parameters provide relevant details such as molecular weight, partition coefficient, oral absorption percentage, properties of Lipinski's (rule of five), and Caco2 cellular permeability (in nm s^−1^). To estimate the absorption and distribution of drugs within the body, the partition coefficient (QP log *P*_0_/*w*) range from −2.0 to 6.5 for every ten molecules. An important factor governing drug metabolism and access to biological membranes is Caco2 cell permeability (QPPCaco), which is observed between 128.256 and 1470.581. All the synthesized compound's access to the central nervous system (CNS) is determined by the blood/brain coefficients (*Q* log *B*/*B*). Based on the predicted permeability of Madin canine kidney cells (QPPMDCK), studies reported that ligands with a <25 nm s^−1^ value have poor permeability, whereas ligands with a >500 nm s^−1^ value have a great deal of potential to be drug-like molecules. The overall human oral absorption percentage for compounds ranged from 85.8 to 100%. PSA describes a molecule's ability to form hydrogen bonds. In humans, this information correlates with the number of drugs absorbed through the intestinal tract. PSA > 140 Å indicates poor absorption of a molecule. There are 95% of synthesized compounds with PSA < 140 Å, which indicates that these molecules are well absorbed by the digestive system. All these pharmacokinetic parameters were found within the acceptable range defined for human use except acarbose (standard) as shown in Table 4. Based on this, we analyzed ten compounds from the most active to the least active molecule as shown in Table 4, indicating that they are possessing drug-like molecules.

**Table 4:** ADME properties of compounds (5a–5j) using QikProp^a^

Comp.	HB donor	HB acceptor	QP log *P*_0_/*w*	QPPCaco	QP log *B*/*B*	QPPMDCK	PHOA	PSA	Rule of five
Acceptable range	≤5	≤10	−2 to 6.5	25 poor; >500 great	−3 to 1.2	<25 poor; >500 great	>80% high; <25% low	≤140	≤5
5a	0	5.5	2.556	631.261	−0.29	2936.205	92.029	95.604	0
5b	0	5.5	2.13	649.365	−0.414	1318.433	89.758	95.386	0
5c	1.5	6.5	1.045	136.105	−1.292	108.943	71.259	124.17	0
5d	1	6.25	1.234	157.879	−1.23	127.894	73.516	120.092	0
5e	0	5.5	1.585	524.643	−0.645	468.378	84.904	97.801	0
5f	0	6.25	1.642	520.906	−0.746	464.759	85.182	105.819	0
5g	0	5.5	2.09	524.617	−0.501	1156.401	87.866	97.786	0
5h	0	6.5	1.461	75.543	−1.456	119.714	69.114	140.611	0
5i	0	6.5	0.616	62.767	−1.66	47.194	64.283	142.671	0
5j	2	6.5	1.245	146.285	−1.430	1186.224	90.156	126.268	0
Acarbose	13	32.1	−7.276	0.04	−5.765	0.01	0	317.259	3

a HB donor: hydrogen bond donor; HB acceptor: hydrogen bond acceptor; QPPCaco: predicted apparent Caco-2 cell permeability in nm s^−1^; QP log *B*/*B*: predicted brain/blood partition coefficient; QPPMDCK: predicted apparent MDCK cell permeability; PHOA: percentage of human oral absorption: PSA: predicted polar surface area.

### Drug-likeness prediction using Osiris property

Based on Osiris property explorer predictions, the results of toxicity risks and drug score assessments for synthesized compounds (5a–5j) are shown in Table 5. Using this tool, we can predict compounds based on their functional groups, which are similar to our extensively studied compounds by *in vitro* methods. There are three color codes for the results: red, green, and yellow.^26^ In the green color, there is low toxicity, in the yellow color there are mild toxins and in the red color, there is a high probability of toxicity. The results indicate that all the compounds showing green color would be safe and did not show toxicity concerning tumorigenicity, mutagenicity, irritant effect, and effect on the reproductive system. All the synthesized compounds showed drug-like properties for their drug scores, drug-likeness scores, and other biochemical and toxicity factors. All the synthesized compounds showed an acceptable range of physicochemical and toxicity parameters as shown in Table 5.

**Table 5:** Drug likeness/scores and toxicity calculation of compounds (5a–5j) based on Osiris property explorer

Compounds	Drug-relevant properties				Toxicity			
	*C* log *P*	Solubility	Drug likeness	Drug score	Tumorigenic	Reproductive effect	Irritant effect	Mutagenicity
5a	1.97	−4.76	4.94	0.91	Green	Green	Green	Green
5b	1.49	−4.13	−0.47	0.63	Green	Green	Green	Green
5c	0.08	−3.37	2.00	0.90	Green	Green	Green	Green
5d	0.42	−3.00	2.05	0.90	Green	Green	Green	Green
5e	0.76	−3.29	3.54	0.95	Green	Green	Green	Green
5f	0.69	−3.31	−0.72	0.63	Green	Green	Green	Green
5g	1.37	−4.03	3.50	0.93	Green	Green	Green	Green
5h	0.45	−4.49	−3.21	0.48	Green	Green	Green	Green
5i	−0.16	−3.75	−8.37	0.47	Green	Green	Green	Green
5j	0.07	−2.70	3.16	0.93	Green	Green	Green	Green
Acarbose	−7.18	0.59	−7.40	0.29	Green	Green	Green	Green

### *In vitro* antidiabetic activity

All the newly synthesized compounds (5a–5j) were subjected to an *in vitro* antidiabetic assay by using α-amylase and α-glucosidase methods. The testing of the synthesized derivatives was carried out at a concentration range of 10–50 μg mL^−1^. Acarbose was used as a standard drug. The results are shown in Table 6.

**Table 6:** *In vitro* antidiabetic activity of compounds (5a–5j)^a^

Compounds	IC_50_ values	
	α-Amylase (μM)	α-Glucosidase (μM)
5a	18.61 ± 0.22	17.58 ± 0.19
5b	22.60 ± 0.34	22.25 ± 0.22
5c	34.08 ± 0.48	27.25 ± 0.23
5d	55.43 ± 0.66	45.28 ± 0.79
5e	32.43 ± 0.46	33.52 ± 0.45
5f	39.23 ± 0.52	51.28 ± 0.83
5g	28.87 ± 0.36	27.25 ± 0.24
5h	23.19 ± 0.31	28.25 ± 0.31
5i	21.12 ± 0.23	25.23 ± 0.28
5j	18.42 ± 0.21	17.21 ± 0.22
Acarbose	24.35 ± 1.44	23.73 ± 1.22

a All results expressed are the mean of three individual replicates (*n* = 3, ±SD).

In the α-amylase assay, some of the tested compounds like 5a, 5c, 5h, 5i, and 5j showed very potent activity with IC_50_ values of 18.61 ± 0.22 μM, 22.60 ± 0.48 μM, 23.19 ± 0.31 μM, 21.12 ± 0.23 μM, 18.42 ± 0.21 μM respectively, when compared to the standard acarbose with IC_50_ value 24.35 ± 1.44 μM. In general, the compounds with the presence of electron-withdrawing groups are showing the highest potent activity. However, it is also observed that compounds like 5b (IC_50_ value 34.08 ± 0.48 μM) and 5f (IC_50_ value 39.23 ± 0.52 μM) also displayed moderate to weak activity, due to the presence of electron-donating groups on the aromatic ring.

In the α-glucosidase assay, the results revealed that the tested compounds like 5a, 5b, and 5j displayed very potent inhibitory activity with IC_50_ values in the range of 17.58 ± 0.19 μM, 22.25 ± 0.22 μM, 17.21 ± 0.22 μM respectively when compared to the standard acarbose with IC_50_ value 23.73 ± 1.22 μM. The presence of electron-withdrawing groups (chloro, bromo) on the aromatic ring is showing the highest potent activity. However, a decrease in the activity was also observed in the compounds like 5d (IC_50_ value 45.28 ± 0.79 μM) and 5f (IC_50_ value 51.28 ± 0.83 μM) due to the presence of electron-donating groups (hydroxyl, methoxy). The presence of the phenyl group showed a very moderate activity by the compound 5e (IC_50_ value 33.52 ± 0.45 μM).

### *In vivo* study

Drosophila flies were treated with three synthesized compounds, namely 5a, 5b, and 5j based on *in vitro* screening for enzyme inhibition and docking analysis.^34^ As a positive control, acarbose was used. According to the glucose estimation in the fly whole-body homogenates, acarbose-supplemented groups had significantly lowered glucose levels in HSD along with acarbose groups. The reduction in glucose level was found to be about 62%. Similar to acarbose, the synthesized compounds also showed a significant reduction in glucose levels of both male and female fly groups fed on HSD with synthesized compounds (5a, 5b, and 5j).

**Fig. 5:**
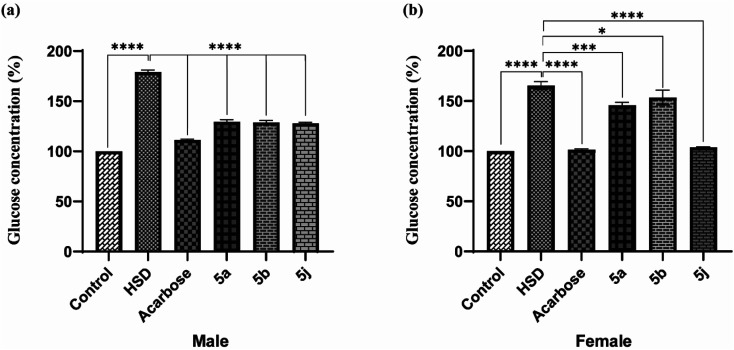
*In vivo* testing for antihyperglycemic effects in *Drosophila melanogaster* ((a) male; (b) female).

The percent reduction in glucose levels was found to be 27.7, 28.4, and 29.05% for 5a, 5b, and 5j respectively in male flies. Whereas the reduction in glucose levels was 12.1, 7.03, and 37% for 5a, 5b, and 5j treated female fly groups (Fig. 5). However, at a given concentration, these tested compounds showed less activity, compared to commercially available acarbose. However, this reduction in antihyperglycemic activity was significant for 5b and 5j whereas, this activity was insignificant for 5a. The antihyperglycemic effects of the tested compounds can be improved by increasing their doses.^35,36^

### Structure–activity relationship

**Fig. 6:**
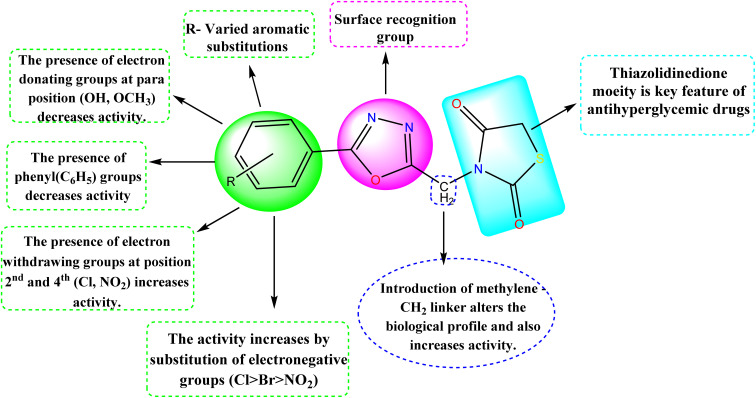
Structure–activity relationship (SAR) of the compounds (5a–5j).

The detailed SAR of the compounds is summarized in Fig. 6. The SAR study reveals, that combination of two pharmacophores *viz.*, thiazolidinedione and 1,3,4-oxadiazole moiety will enhance the diabetic activity by the incorporation of methylene linker.

### MTT assay

**Fig. 7:**
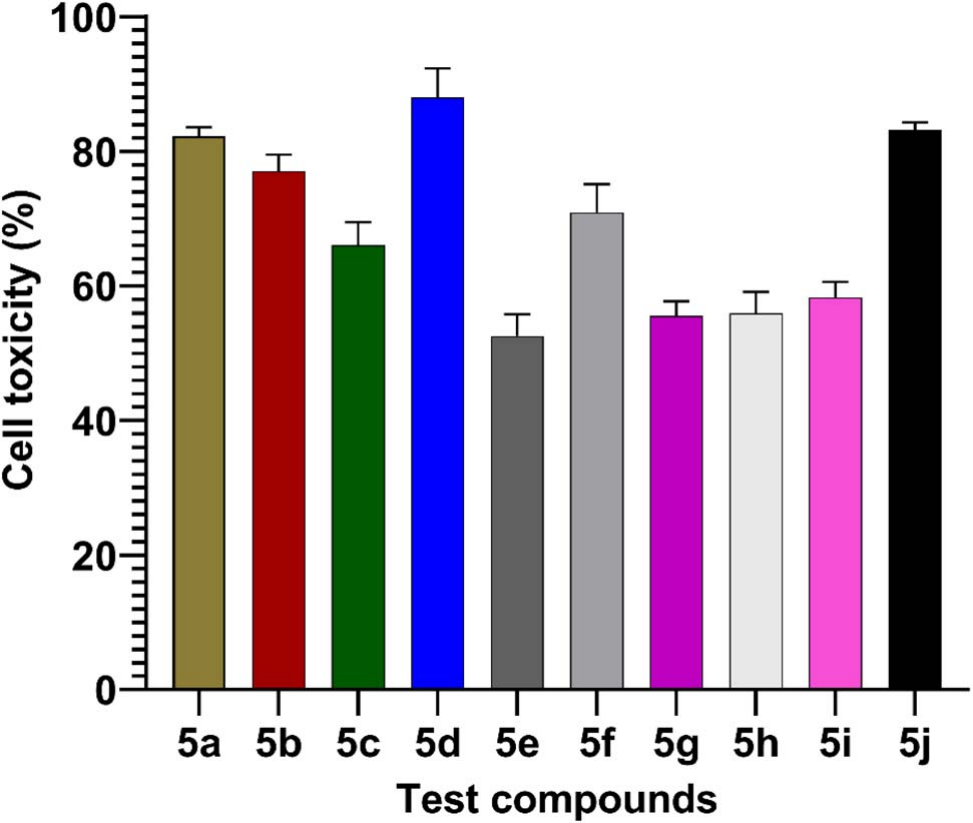
Values are mean ± SD from triplicate determined (*n* = 3). The percentage cell toxicity of different test compounds (5a–5j) at a concentration of 100 μmol.

The MTT assay was used to measure the effects of synthesized compounds on NIH-3T3 cells as shown in Fig. 7. MTT is a yellow water-soluble tetrazolium dye that is reduced by live cells to a purple formazan product, insoluble in aqueous solutions. The amount of formazan generated is directly proportional to the number of viable cells. The synthesized compounds during 24 h of incubation induced 87.4–0% inhibition of cellular viability in comparison to the control. In this study, the highest concentration (100 μmol) was used to calculate percentage inhibition. The compounds 5d (87%) and 5j (83%) showed higher cell toxicity when compared to other tested compounds (Fig. 7).

## Conclusion

This research represents the synthesis of novel series of 3-((5-phenyl-1,3,4-oxadiazol-2-yl)methyl)thiazolidine-2,5-dione derivatives. All the synthesized compounds were confirmed by spectroscopic methods and evaluated for *in vitro* anti-hyperglycemic activity by α-amylase and α-glucosidase inhibition assay. *In vivo*, *Drosophila melanogaster* fly model was used to test the efficacy of these compounds and was raised on a diet containing even concentrations of either acarbose or compounds with most *in vitro* activity, *i.e.*, 5a, 5b, and 5j from the results. There was a significant difference between standard acarbose and the compounds 5a, 5b, and 5j in terms of antihyperglycemic activity. Therefore, clinical research has become increasingly concerned with developing compounds with antihyperglycemic activity. Considering this, it was found that compounds 5a, 5b, and 5j had both these properties. The findings indicated that these compounds might provide a rational approach to the development of novel and potent diabetes genetic enzyme inhibitors. However, the study requires further preclinical trials and these compounds can be used as very good candidates for the effective therapeutic management of diabetes mellitus.

## Author contributions

RBC did the conception of the work, design of the work, experimental work, and the interpretation of data and drafting of the manuscript. MGS and JGP contributed to the design of work, experimental work done, and results in analysis. AP, VP, and SRSB helped in carrying out the design work, and MGS, SDN, and RBC reviewed the manuscript. All authors read and approved the final manuscript.

## Conflicts of interest

There are no competing financial interests or personal relationships influencing the findings of this paper.

## Supplementary Material

RA-013-D2RA07247E-s001
