# Supplementary material for: Novel hybrids of thiazolidinedione-1,3,4-oxadiazole derivatives: synthesis, molecular docking, MD simulations, ADMET study, in vitro, and in vivo anti-diabetic assessment
Source: RSC Adv. 2023 Jan 9;13(3):1567–79. doi: 10.1039/d2ra07247e (PMC9828437; doi:10.1039/d2ra07247e)

Electronic Supplementary Material (ESI) for RSC Advances.
This journal is © The Royal Society of Chemistry 2022

Analytical Supplymentry Data:

3-**((5-(2,4-dichlorophenyl)-1,3,4-oxadiazol-2-yl)methyl)thiazolidine-2,4-dione (5a):** Molecular formula: $C_{12}H_7Cl_2N_3O_3S$, Yellow solid, Yield: 78%, Melting point: 210-212°C; FT-IR (cm-[1]): 3020 (Ar, C-H), 2927 (CH_2), 1734 (C=O), 1585 (C=N), 1513 (C=C). ^{1}H-NMR (400 MHz, DMSO-d_6, δ, ppm): 8.78-7.10 (m, Ar-H, 3H), 4.33 (s, CH_2, 2H), 3.26(s, CH_2, 2H): ^{13}CNMR (100MHz, DMSO-d_6, δ, ppm): 165.3, 164.2, 163.4, 162.2, 135.0, 129.2, 128.6, 123.3, 122.6, 119.7, 46.3, and 39.0: LC-MS (m/z): calculated for $C_{12}H_7Cl_2N_3O_3S$ is 344.1; found: 344.1 (M+).

3-((5-(4-bromophenyl)-1,3,4-oxadiazol-2-yl) methyl) thiazolidine-2,4-dione (5b): Molecular formula: $C_{12}H_8BrN_3O_3S$, Yellow solid, Yield: 87%, Melting point: 185-187 °C; FT-IR (cm-[1]): 3003 (Ar, CH), 2924 (CH_2), 1735(C=O), 1586 (C=N), 1516 (C=C), 742 (C-Br). ^{1}H-NMR (400 MHz, DMSO-d_6, δ, ppm): 7.69 -7.11 (m, Ar-H, 4H), 4.41 (s, CH_2, 2H), 3.36(s, CH_2, 2H),:^{13}CNMR (100MHz, DMSO-d_6, δ, ppm): 165.9, 159.9, 156.9, 149.8, 129.3, 128.7, 128.6, 123.9, 123.4, 119.7, 45.8 and 34.5: LC-MS (m/z) calculated for $C_{12}H_8BrN_3O_3S$ is 354.1; found: 354.1 (M+).

3-((5-(4-aminophenyl)-1,3,4-oxadiazol-2-yl)methyl)thiazolidine-2,4-dioneone (5c): Molecular formula: $C_{12}H_{10}N_4O_3S$, Yellow solid, Yield: 82%, Melting point: 198-200°C; FT-IR (cm-[1]): 3336 (-NH), 3007(Ar, CH), 2932(CH_2), 1712(C=O), 1591 (C=N), 1512(C=C). ^{1}H-NMR (400 MHz, DMSO-d_6, δ, ppm):8.78-8.14 (m, Ar-H, 4H), 7.10(s, NH_2, 2H), 4.98 (s, CH_2, 2H), 3.36(s, CH_2, 2H): ^{13}CNMR (100MHz, DMSO-d_6, δ, ppm): 166.0, 164.6, 163.3, 161.2, 144.8, 135.0, 129.2, 128.6, 123.3, 119.4, 45.4, and 33.6: LC-MS (m/z): calculated for $C_{12}H_{10}N_4O_3S$ is 290.2; found: 290.2 (M+).

3-((5-(4-hydroxyphenyl)-1,3,4-oxadiazol-2-yl)methyl)thiazolidine-2,4-dione (5d): Molecular formula: $C_{12}H_9N_3O_4S$, Yellow solid, Yield: 73%, Melting point: 192-194°C; FT-IR (cm-[1]): 3315 (-OH),): 3020 (Ar,C-H), 2927 (CH_2), 1734 (C=O), 1585 (C=N), 1513 (C=C). ^{1}H-NMR (400 MHz, DMSO-d_6, δ, ppm): 9.41 (s, OH, 1H), 8.19 -7.11 (m, Ar-H, 4H), 4.54 (s, CH_2, 2H), 3.35(s, CH_2, 2H): ^{13}CNMR (100MHz, DMSO-d_6, δ, ppm): 165.4, 164.6, 163.4, 161.7, 149.8, 135.0, 129.3, 128.6, 123.4, 119.7, 45.4 and 34.6: LC-MS (m/z): calculated for $C_{12}H_9N_3O_4S$ is 291.2; found: 291.2 (M+).

3-((5-phenyl-1,3,4-oxadiazol-2-yl)methyl)thiazolidine-2,4-dione (5e):

Molecular formula: $C_{12}H_9N_3O_3S$, Yellow solid, Yield: 69%, Melting point: 215-217°C; FT-IR (cm^{-1}): 3017 (Ar, CH), 2857 (CH_2), 1736 (C=O), 1598 (C=N), 1515 (C=C). ^{1}H-NMR (400 MHz, DMSO- d_6, δ, ppm): 8.40-7.11(m, Ar-H, 5H)3.35 (s, CH_2, 2H), 3.05(s, CH_2, 2H): ^{13}CNMR (100MHz, DMSO-d_6, δ, ppm): 167.0, 165.7, 163.2, 162.1, 131.5, 129.3, 128.6, 124.4, 123.3, 119.7, 45.8, and 35.2: LC-MS (m/z): calculated for $C_{12}H_9N_3O_3S$ is 275.2; found: 275.2 (M+).

3-((5-(4-methoxyphenyl)-1,3,4-oxadiazol-2-yl)methyl)thiazolidine-2,4-dione (5f): Molecular formula: $C_{13}H_{11}N_3O_4S$, Yellow solid, Yield: 86%, Melting point: 178-180°C; FT-IR (cm^{-1}): 3046 (Ar, CH), 2896 (CH_2), 1687(C=O), 1584 (C=N), 1515 (C=C). ^{1}H-NMR (400 MHz, DMSO-d_6, δ, ppm): 8.16-7.07 (m, Ar-H, 4H), 4.34 (s, CH_2, 2H), 3.83 (s, OCH_3, 3H), 3.32 (s, CH_2, 2H): ^{13}CNMR (100MHz, DMSO-d_6, δ, ppm): 168.8, 166.2, 165.1, 163.0, 161.8, 128.5, 124.0, 123.3, 119.7, 53.4, 45.4 and 33.6: LC-MS (m/z): calculated for $C_{13}H_{11}N_3O_4S$ is 305.3; found: 305.3 (M+).

3-((5-(4-chlorophenyl)-1,3,4-oxadiazol-2-yl)methyl)thiazolidine-2,4-dione (5g):

Molecular formula: $C_{12}H_8ClN_3O_3S$, Yellow solid, Yield: 81%, Melting point: 172-174°C; FT-IR (cm^{-1}): 3035 (Ar, CH), 2920 (CH_2), 1731(C=O), 1592 (C=N), 1517(C=C). ^{1}H-NMR (400 MHz, DMSO-d_6, δ, ppm): 8.16-7.09 (m, Ar-H, 4H), 3.89 (s, CH_2, 2H), 3.38(s, CH_2, 2H): ^{13}CNMR (100MHz, DMSO-d_6, δ, ppm): 167.9, 164.7, 162.2, 161.7, 142.3, 135.0, 129.2, 128.6, 123.3, 119.7, 46.7, and 34.4: LC-MS (m/z): calculated for $C_{12}H_8ClN_3O_3S$ is 309.7; found: 309.3 (M+).

3-((5-(2-chloro-4-nitrophenyl)-1,3,4-oxadiazol-2-yl)methyl)thiazolidine-2,4-dione (5h): Molecular formula: $C_{12}H_7ClN_4O_5S$, Yellow solid, Yield:74%, Melting point: 161-163°C; FT-IR (cm^{-1}): 3046 (Ar, CH), 2924 (CH_2), 1734 (C=O), 1585 (C=N), 1514 (C=C). ^{1}H-NMR (400 MHz, DMSO-d_6, δ, ppm): 8.19-7.11 (m, Ar-H, 3H), 4.14 (s, CH_2, 2H), 3.36(s, CH_2, 2H): ^{13}CNMR (100MHz, DMSO-d_6, δ, ppm): 165.9, 164.3, 161.9, 161.0, 149.8, 135.0, 129.3, 128.6, 123.4, 119.7, 44.8 and 34.5: LC-MS (m/z): calculated for $C_{12}H_7ClN_4O_5S$ is 354.7; found: 354.7 (M+).

3-((5-(4-nitrophenyl)-1,3,4-oxadiazol-2-yl)methyl)thiazolidine-2,4-dione(5i): Molecular formula: $C_{12}H_8N_4O_5S$, Yellow solid, Yield: 81%, Melting point: 153-155°C; FT-IR (cm^{-1}): 3005 (Ar, CH), 2955 (CH_2), 1735 (C=O), 1582 (C=N), 1514 (C=C). ^{1}H-NMR (400 MHz, DMSO-d_6, δ, ppm): 8.12-7.69 (m, Ar-H, 4H), 3.83 (s, CH_2, 2H), 3.02(s, CH_2, 2H): ^{13}CNMR

(100MHz, DMSO-d_6, δ, ppm): 164.9, 164.0, 163.2, 162.1, 149.2, 135.105, 129.3, 128.6, 123.4, 119.7, 44.7, and 34.4: LC-MS (m/z): calculated for $C_{12}H_8N_4O_5S$ is 320.2795; found: 320.2735 (M+).

3-((5-(2,4-dihydroxyphenyl)-1,3,4-oxadiazol-2-yl)methyl)thiazolidine-2,4-dione (5j): Molecular formula: $C_{12}H_9N_3O_5S$, Yellow solid, Yield: 73%, Melting point: 204-206°C; FT-IR (cm^{-1}): 3326 (-OH), 3005 (Ar, CH), 2955 (CH_2), 1769 (C=O), 1591 (C=N), 1521 (C=C). ^{1}H-NMR (400 MHz, DMSO-d_6, δ, ppm: 10.14 (s, OH, 2H), 7.92-7.09 (m, Ar-H, 4H), 4.43 (s, CH_2, 2H), 3.32 (s, CH_2, 2H):^{13}CNMR (100MHz, DMSO-d_6, δ, ppm): 165.5, 164.6, 161.4, 149.3, 135.1, 130.4, 128.3, 122.9, 119.6, 45.4 and 34.5: LC-MS (m/z): calculated for $C_{12}H_9N_3O_5S$ is 307.2; found: 307.2 (M+).

Compound 5a : 3-((5-(2,4-dichlorophenyl)-1,3,4-oxadiazol-2-yl)methyl)thiazolidine-2,4-dione

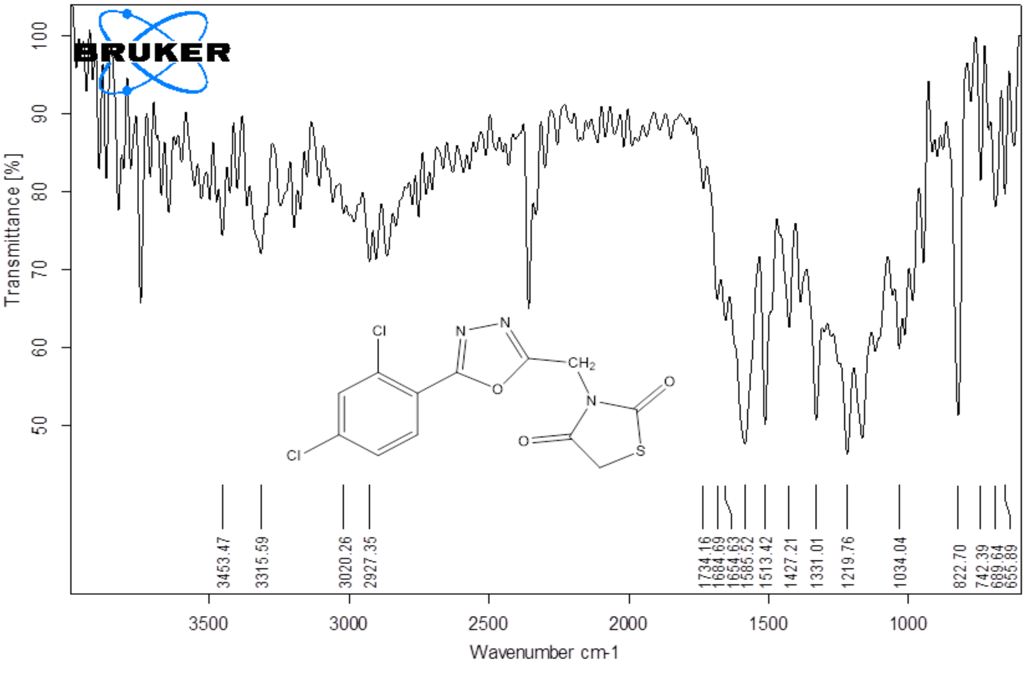

Compound 5a : 3-((5-(2,4-dichlorophenyl)-1,3,4-oxadiazol-2-yl)methyl)thiazolidine-2,4-dione:

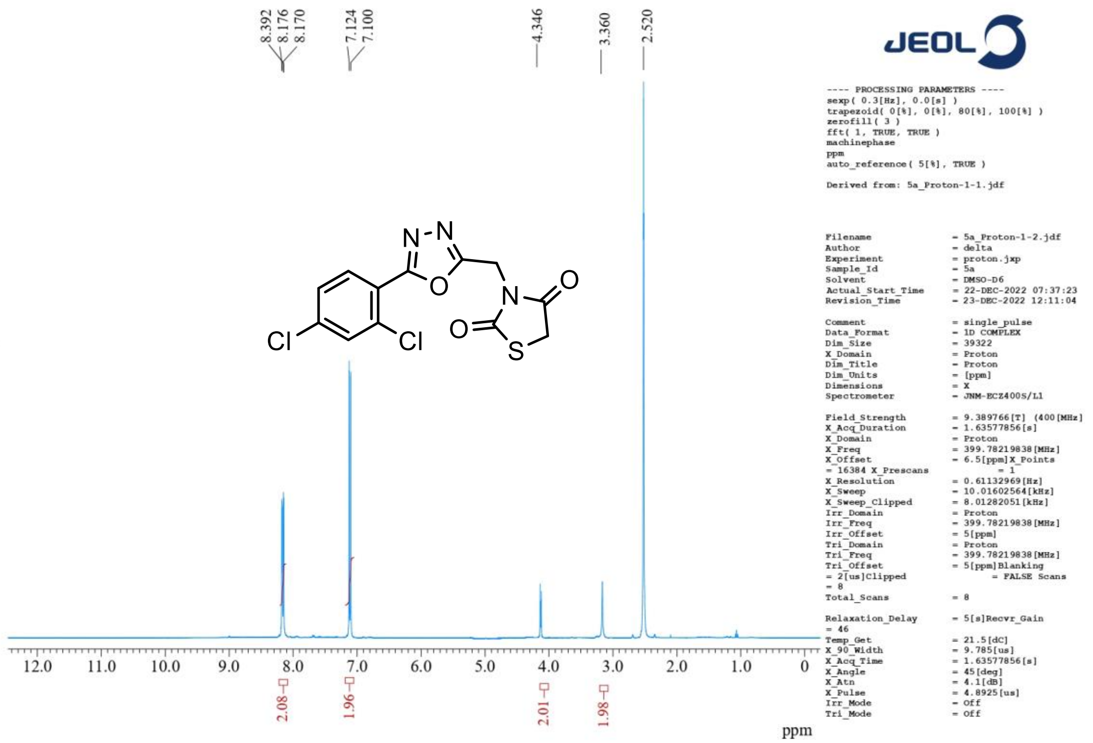

Compound 5a : 3-((5-(2,4-dichlorophenyl)-1,3,4-oxadiazol-2-yl)methyl)thiazolidine-2,4-dione

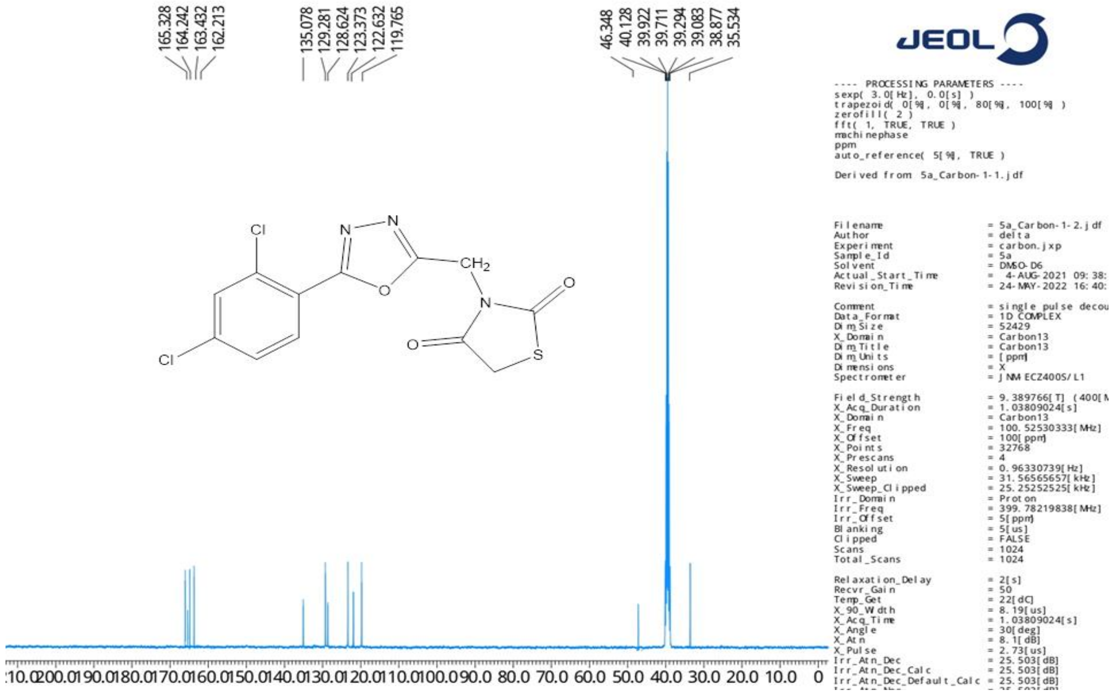

Compound 5a : 3-((5-(2,4-dichlorophenyl)-1,3,4-oxadiazol-2-yl)methyl)thiazolidine-2,4-dione

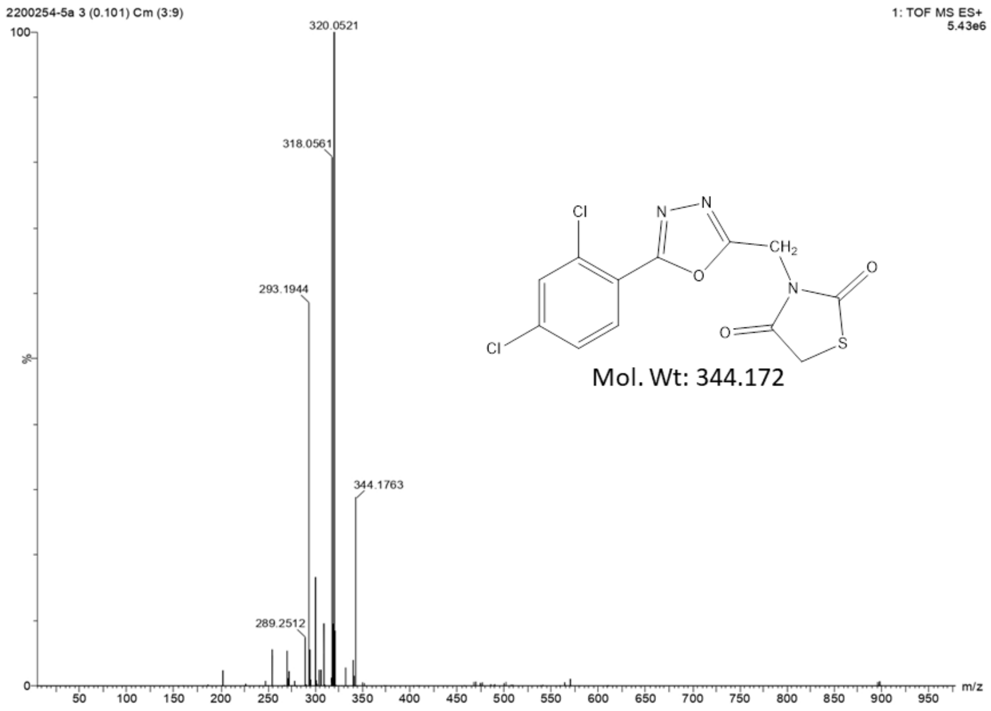

Compound 5b: 3-((5-(4-bromophenyl)-1,3,4-oxadiazol-2-yl)methyl)thiazolidine-2,4-dione

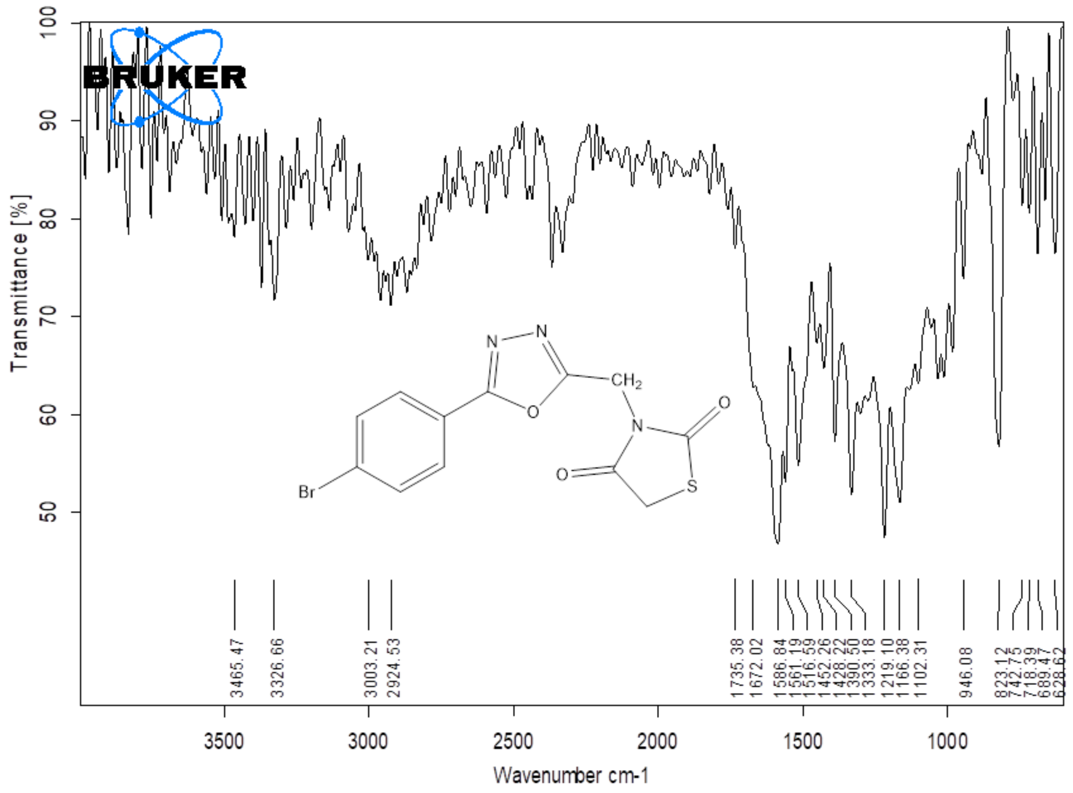

Compound 5b: 3-((5-(4-bromophenyl)-1,3,4-oxadiazol-2-yl)methyl)thiazolidine-2,4-dione

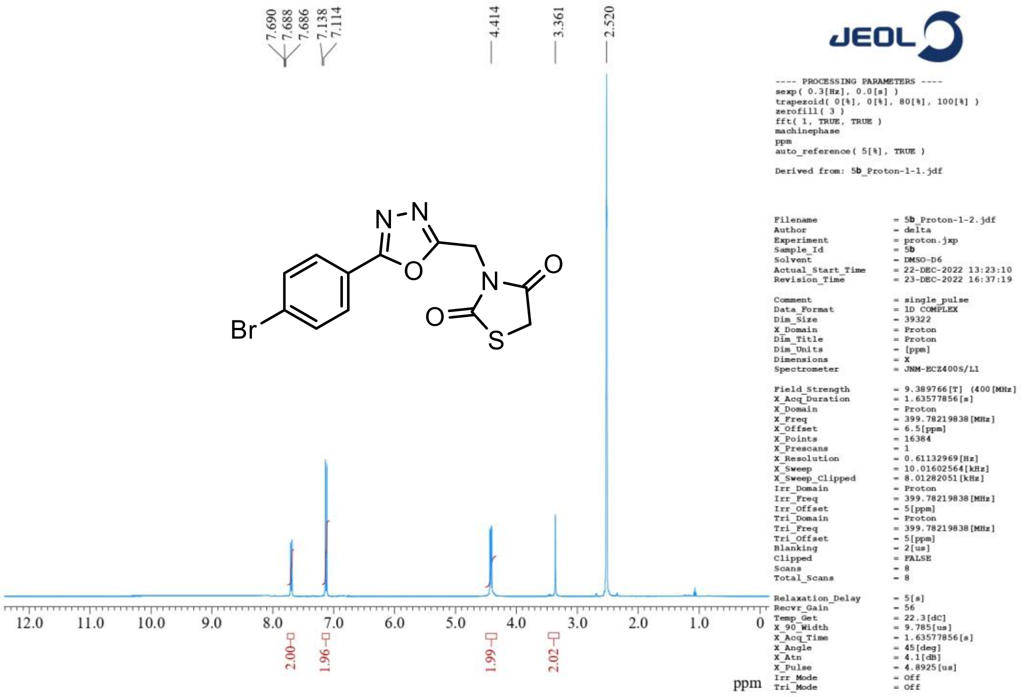

Compound 5b: 3-((5-(4-bromophenyl)-1,3,4-oxadiazol-2-yl)methyl)thiazolidine-2,4-dione

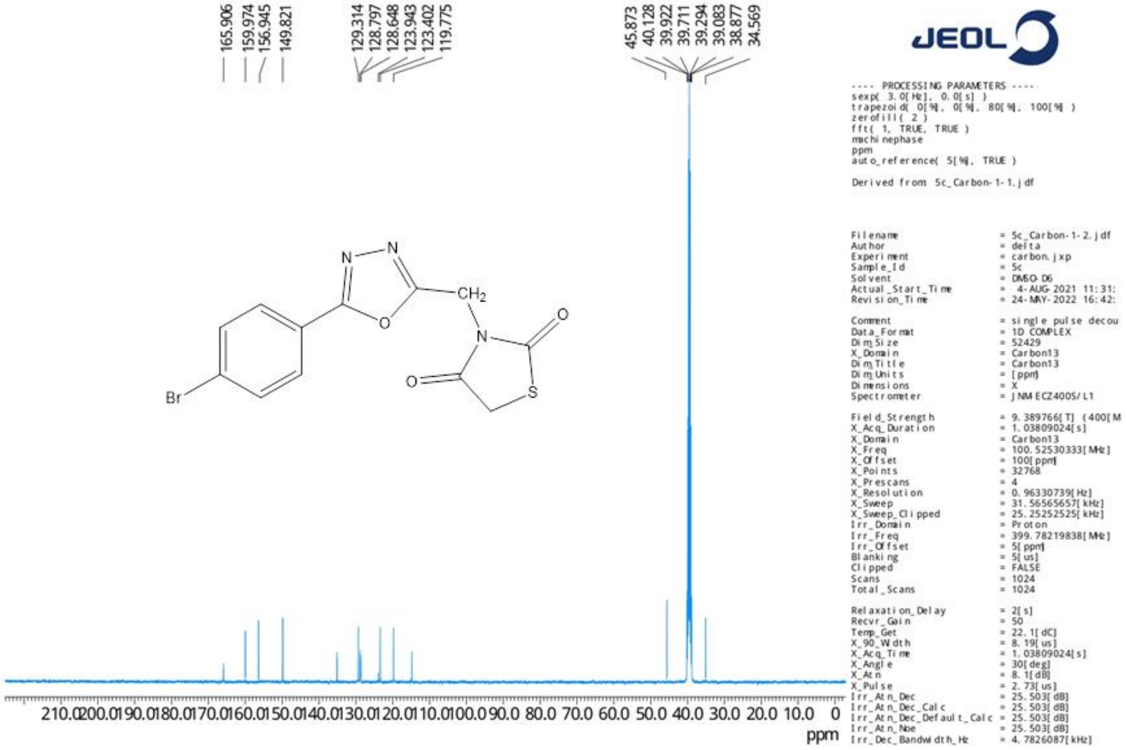

Compound 5b: 3-((5-(4-bromophenyl)-1,3,4-oxadiazol-2-yl)methyl)thiazolidine-2,4-dione

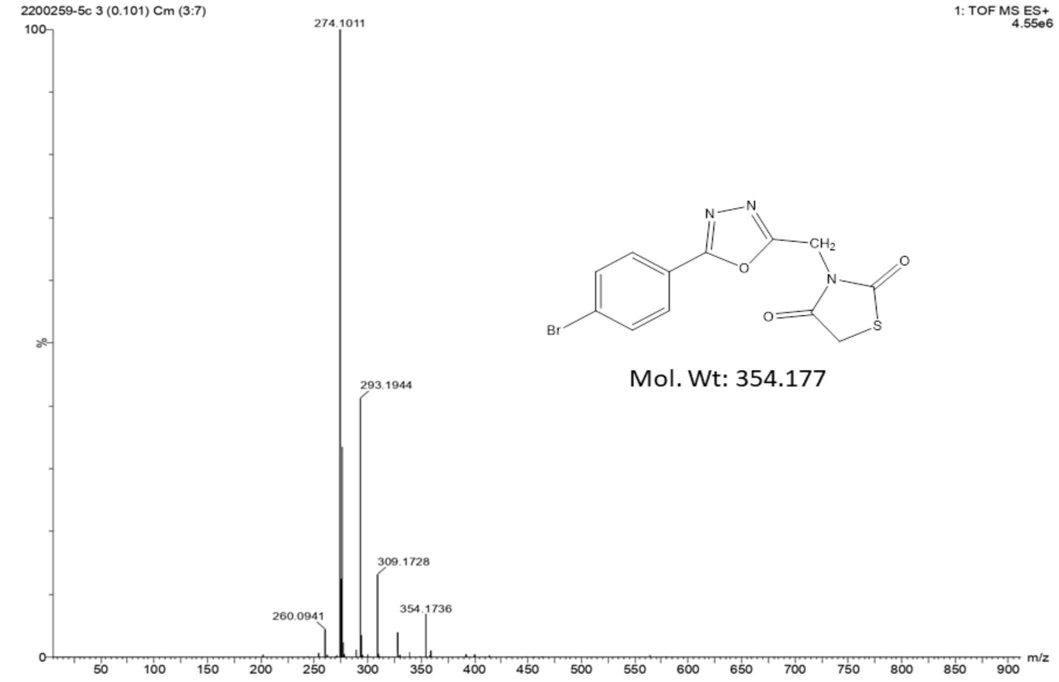

Compound 5c: 3-((5-(4-aminophenyl)-1,3,4-oxadiazol-2-yl)methyl)thiazolidine-2,4-dione

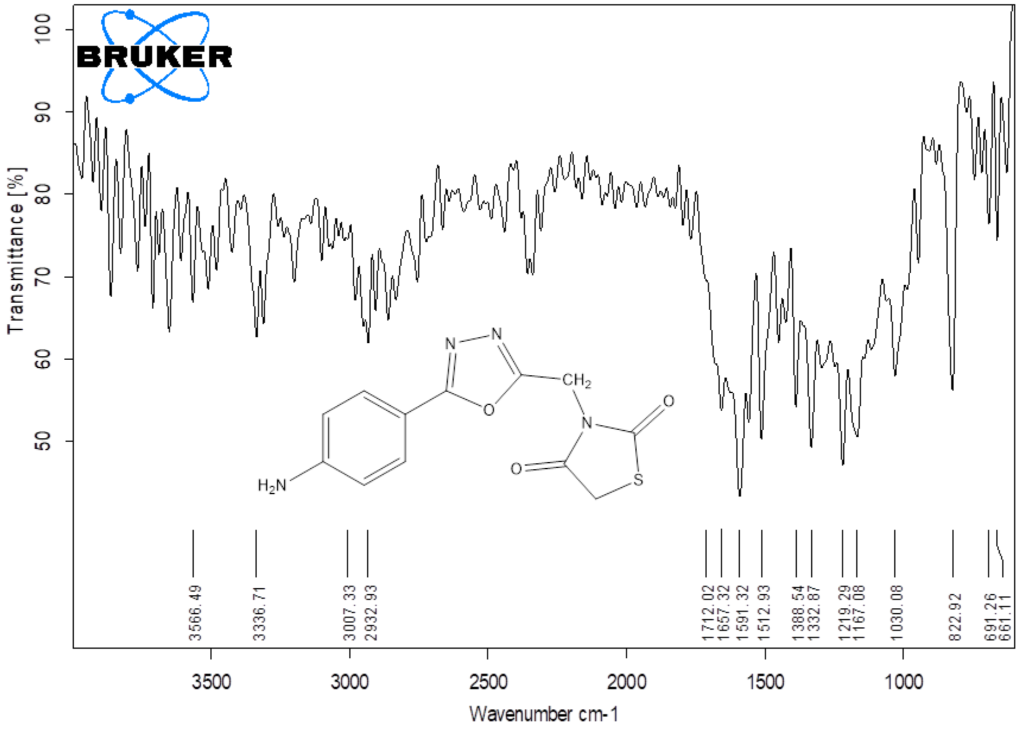

Compound 5c: 3-((5-(4-aminophenyl)-1,3,4-oxadiazol-2-yl)methyl)thiazolidine-2,4-dione

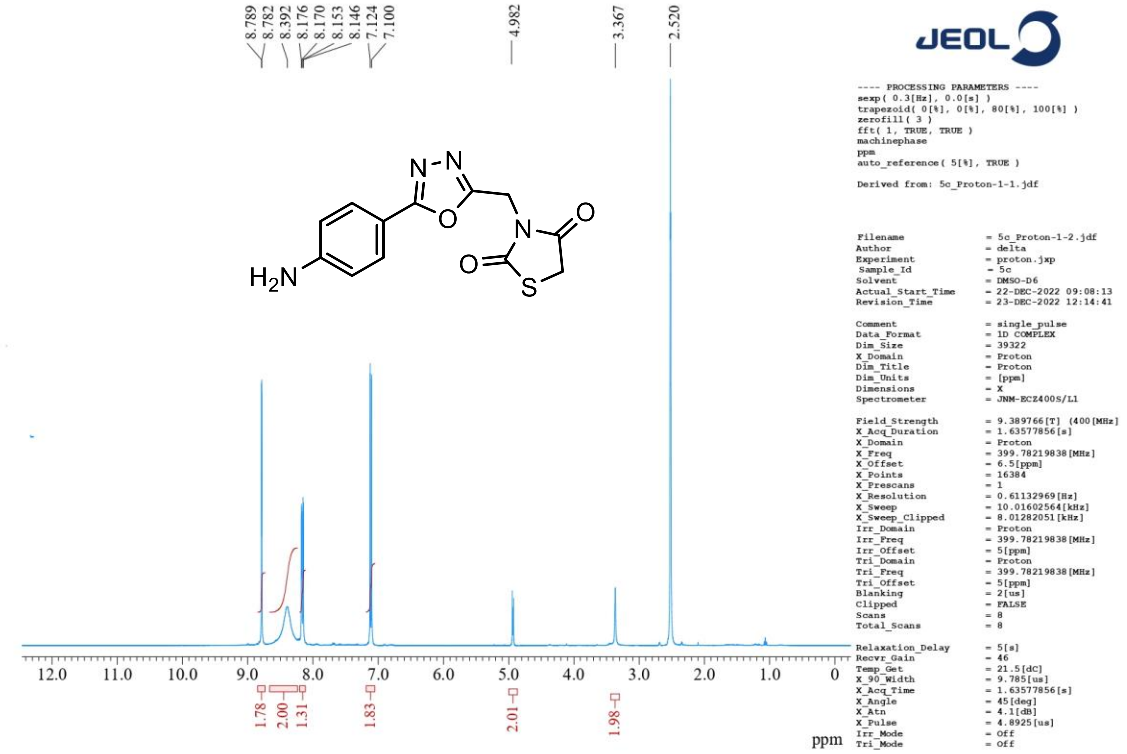

Compound 5c: 3-((5-(4-aminophenyl)-1,3,4-oxadiazol-2-yl)methyl)thiazolidine-2,4-dione

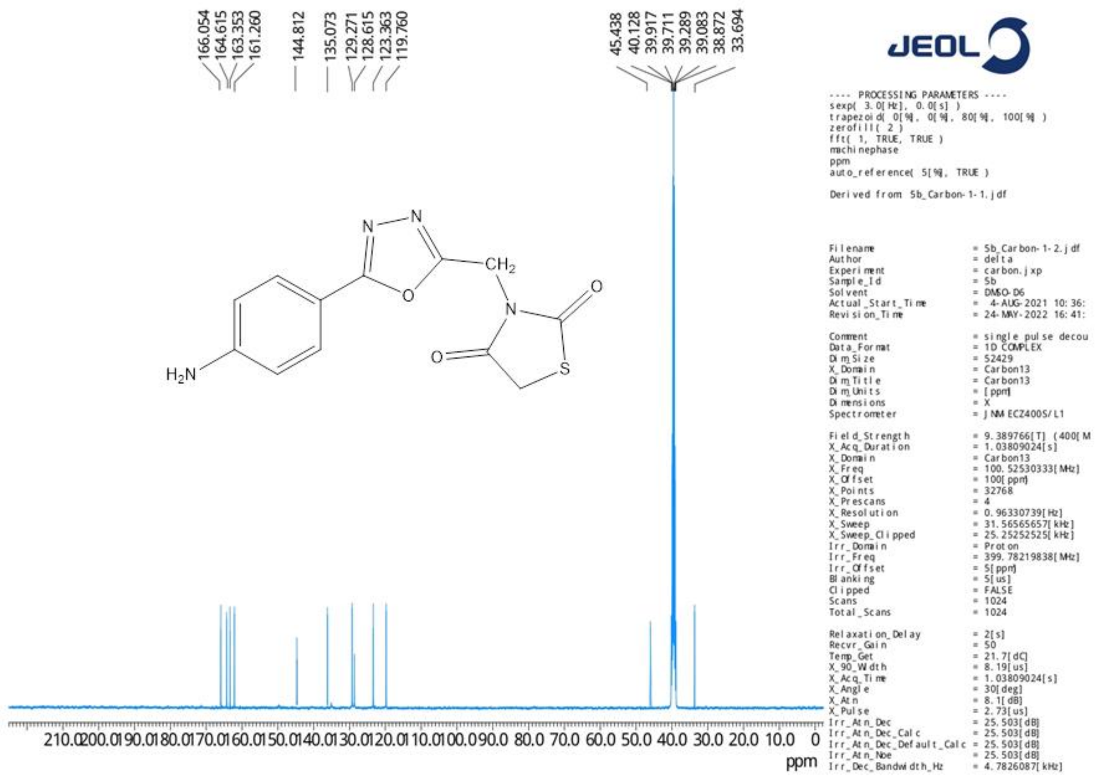

Compound 5c: 3-((5-(4-aminophenyl)-1,3,4-oxadiazol-2-yl)methyl)thiazolidine-2,4-dione

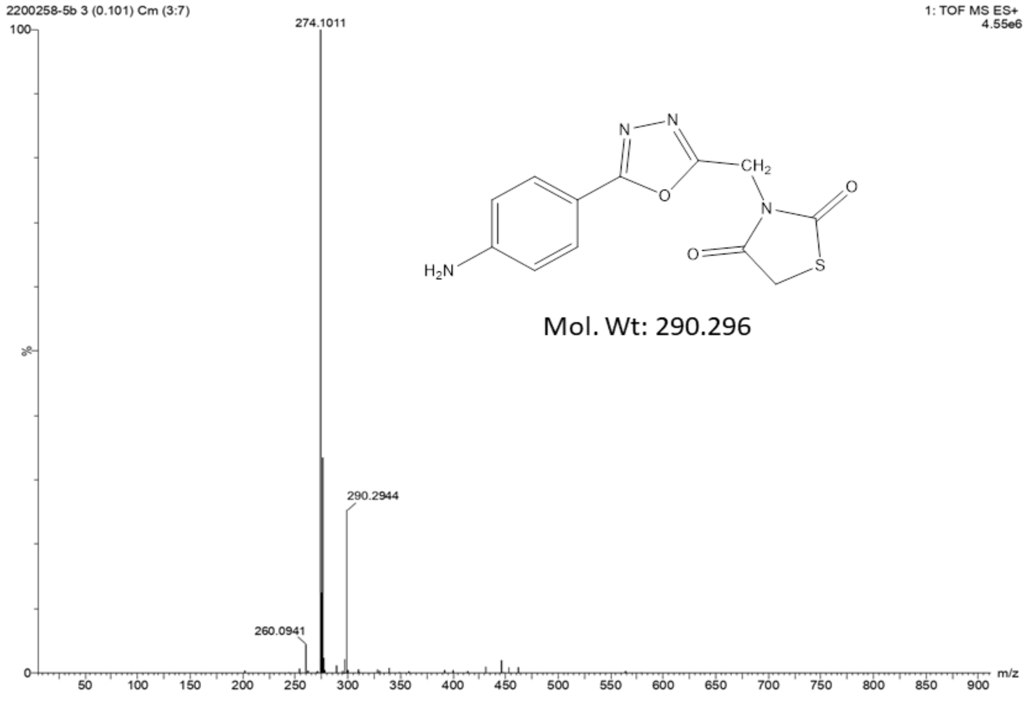

Compound 5d: 3-((5-(4-hydroxyphenyl)-1,3,4-oxadiazol-2-yl)methyl)thiazolidine-2,4-dione:

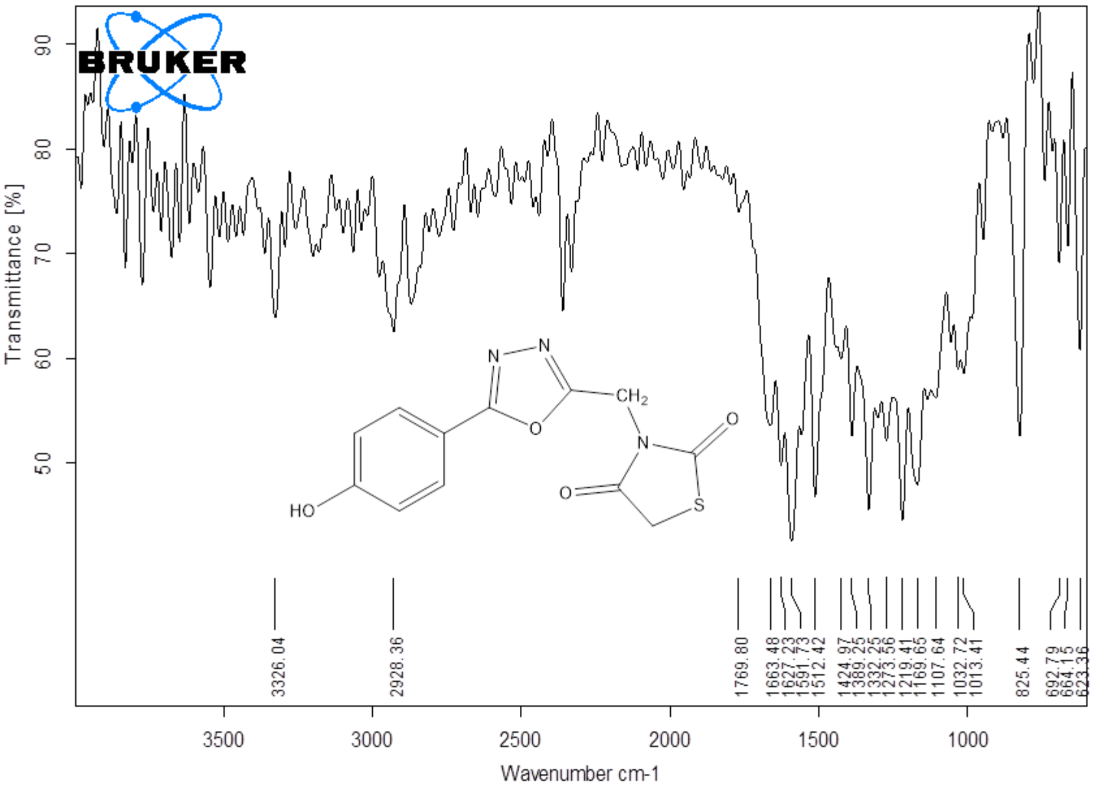

Compound 5d: 3-((5-(4-hydroxyphenyl)-1,3,4-oxadiazol-2-yl)methyl)thiazolidine-2,4-dione

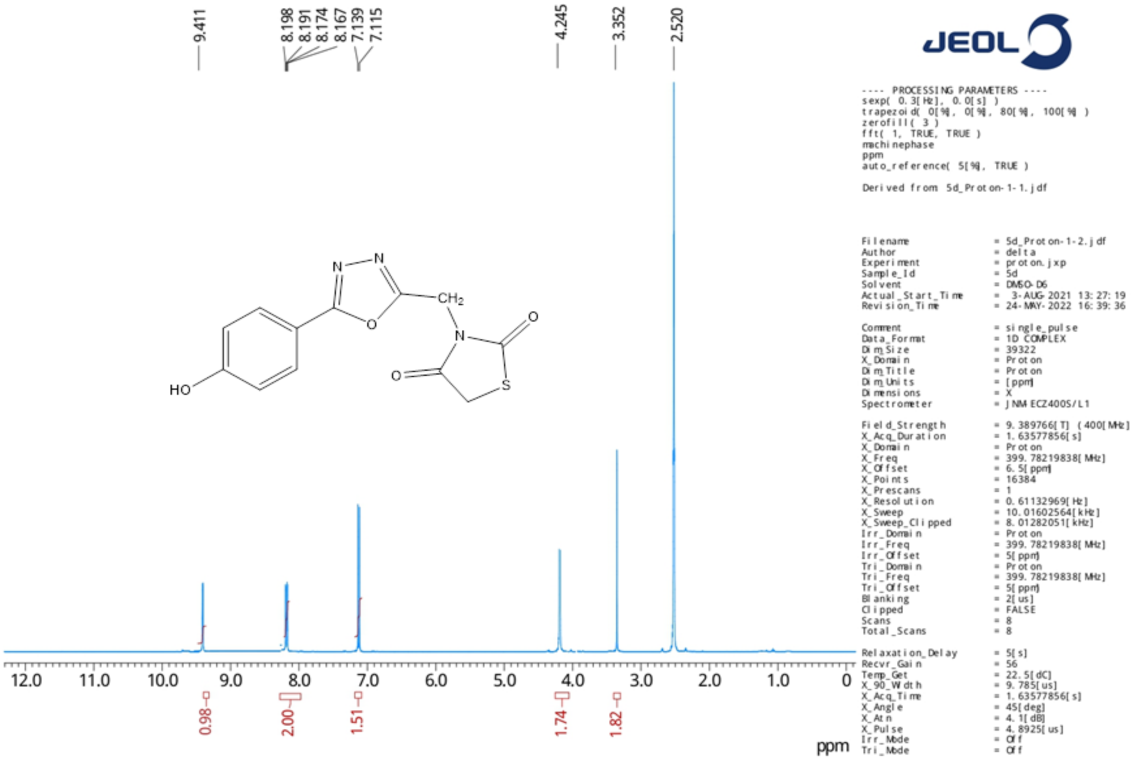

Compound 5d: 3-((5-(4-hydroxyphenyl)-1,3,4-oxadiazol-2-yl)methyl)thiazolidine-2,4-dione

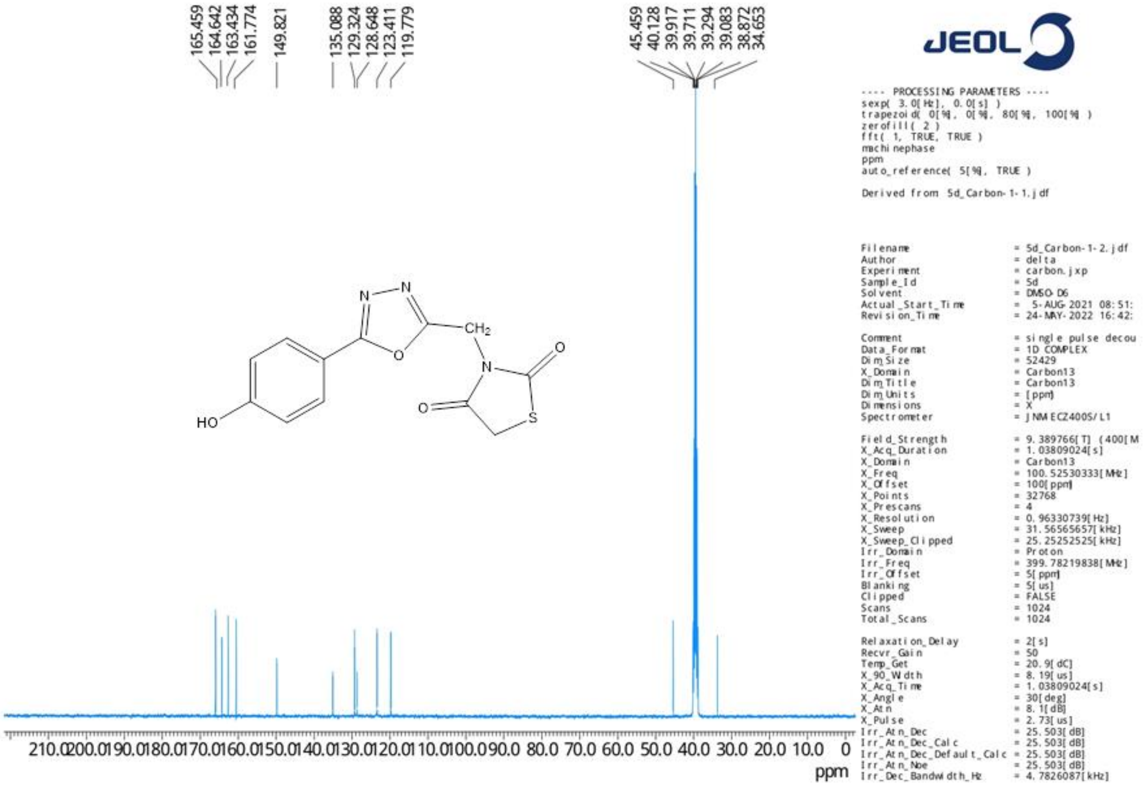

Compound 5d: 3-((5-(4-hydroxyphenyl)-1,3,4-oxadiazol-2-yl)methyl)thiazolidine-2,4-dione

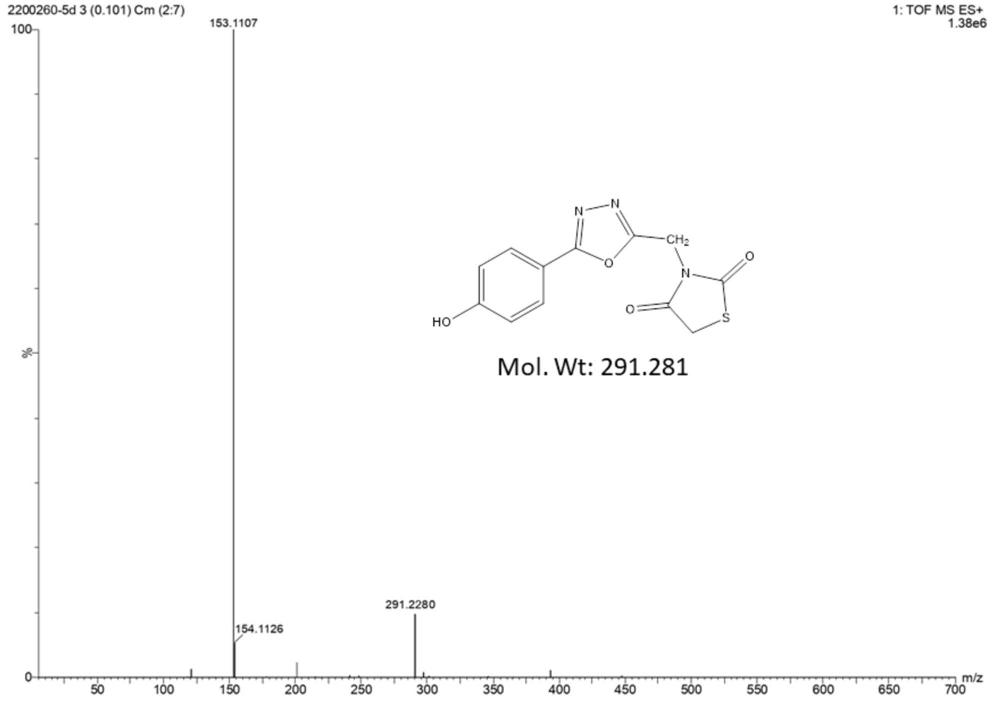

Compound 5e: 3-((5-phenyl-1,3,4-oxadiazol-2-yl)methyl)thiazolidine-2,4-dione:

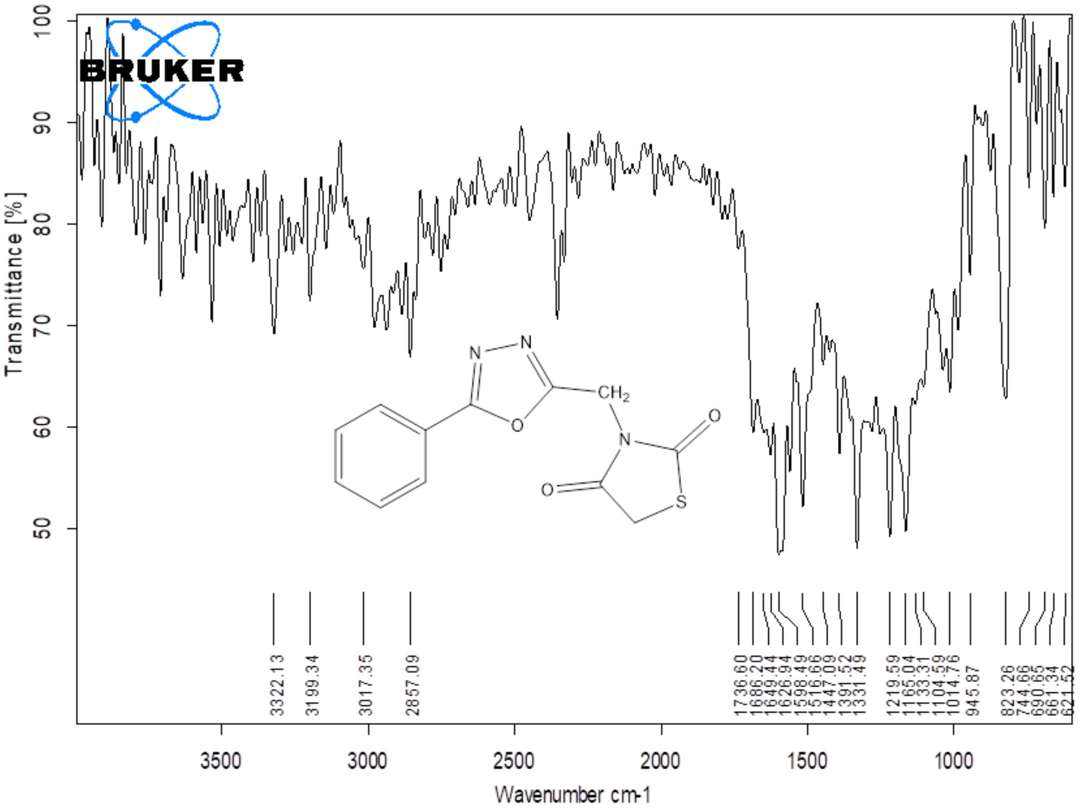

Compound 5e: 3-((5-phenyl-1,3,4-oxadiazol-2-yl)methyl)thiazolidine-2,4-dione:

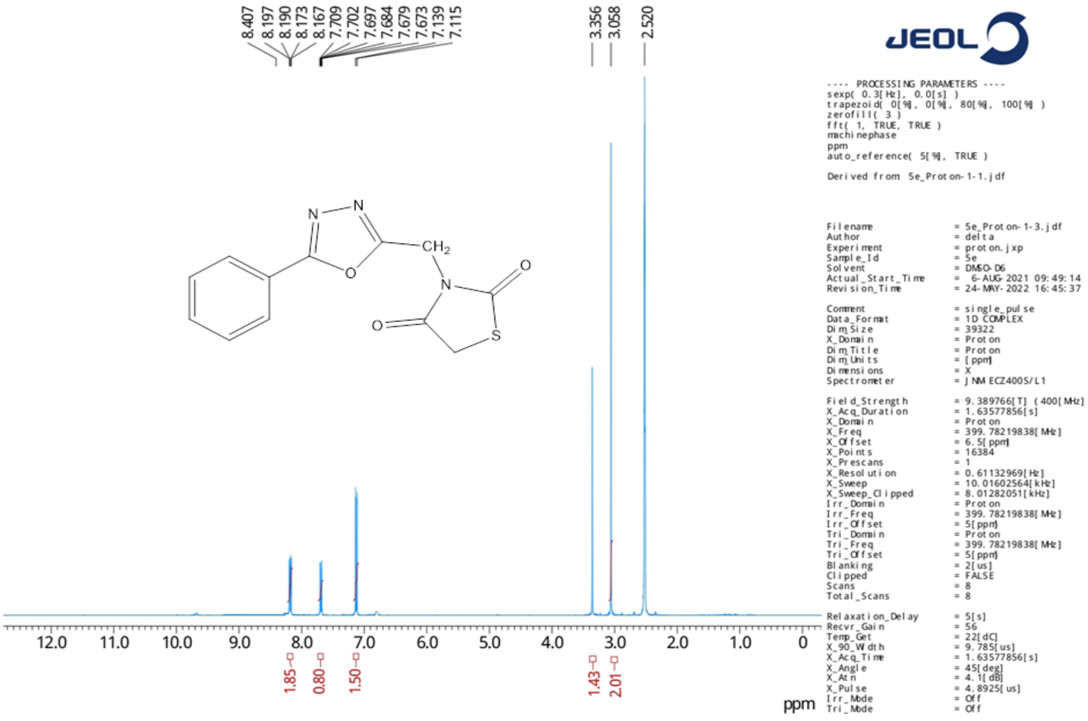

Compound 5e: 3-((5-phenyl-1,3,4-oxadiazol-2-yl)methyl)thiazolidine-2,4-dione:

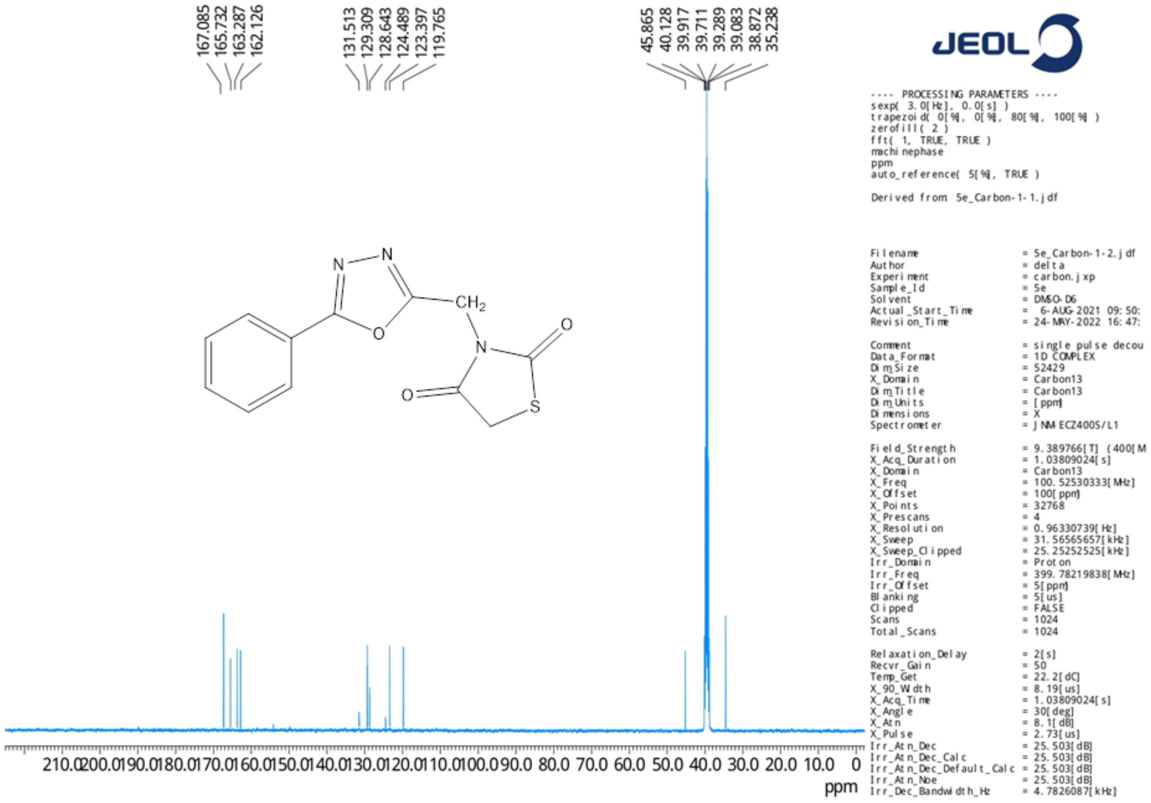

Compound 5e: 3-((5-phenyl-1,3,4-oxadiazol-2-yl)methyl)thiazolidine-2,4-dione:

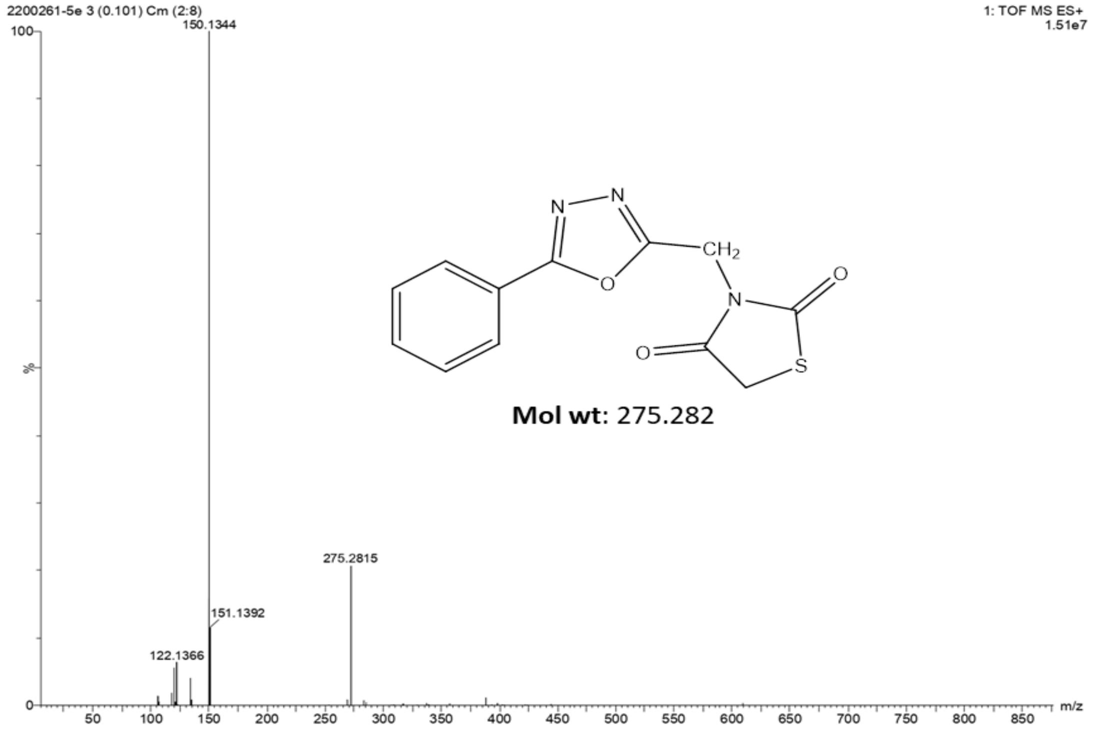

Compound 5f: 3-((5-(4-methoxyphenyl)-1,3,4-oxadiazol-2-yl)methyl)thiazolidine-2,4-dione

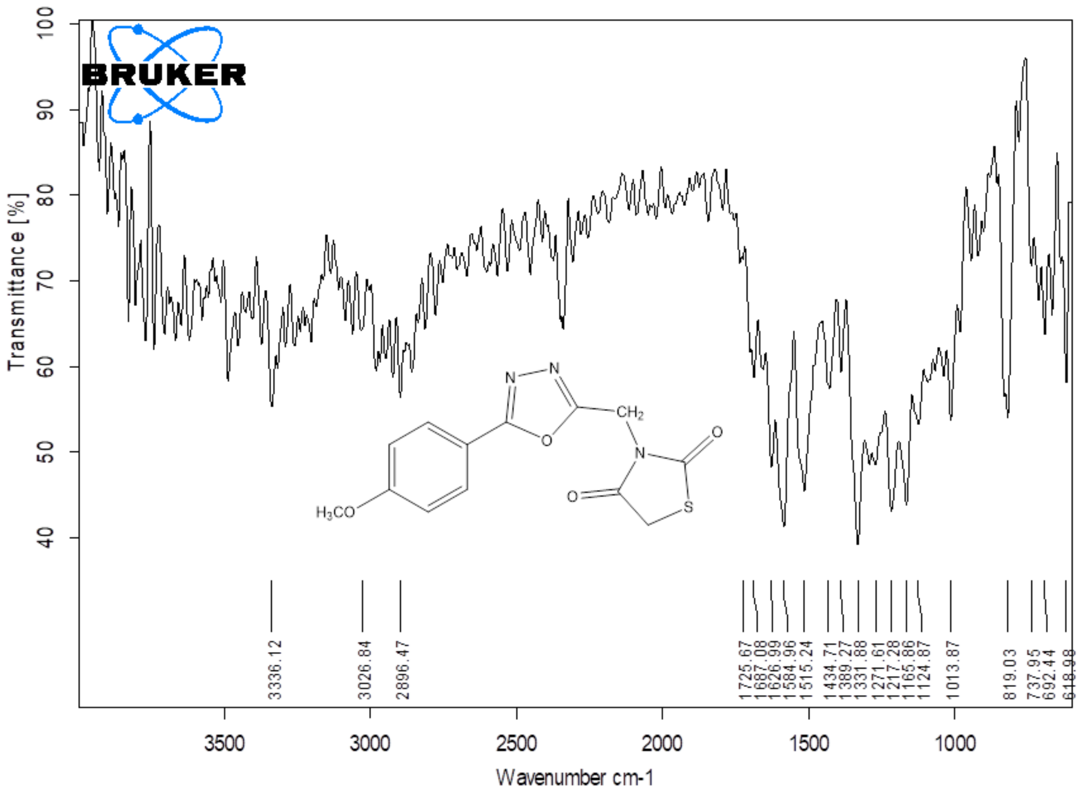

Compound 5f: 3-((5-(4-methoxyphenyl)-1,3,4-oxadiazol-2-yl)methyl)thiazolidine-2,4-dione

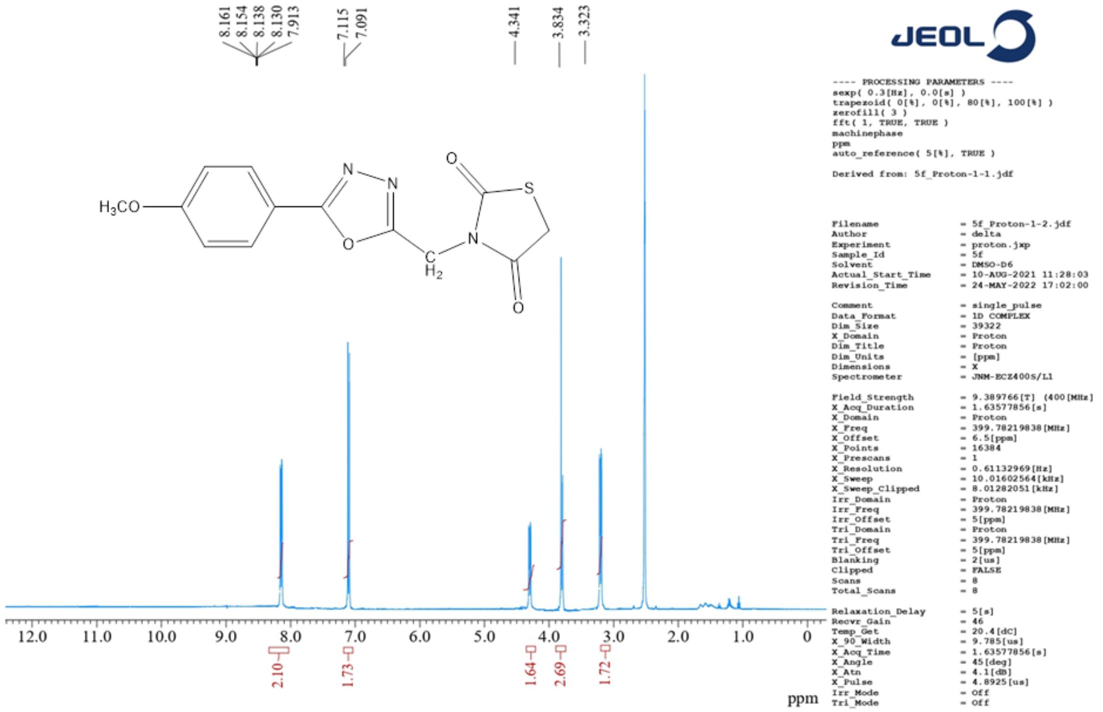

Compound 5f: 3-((5-(4-methoxyphenyl)-1,3,4-oxadiazol-2-yl)methyl)thiazolidine-2,4-dione

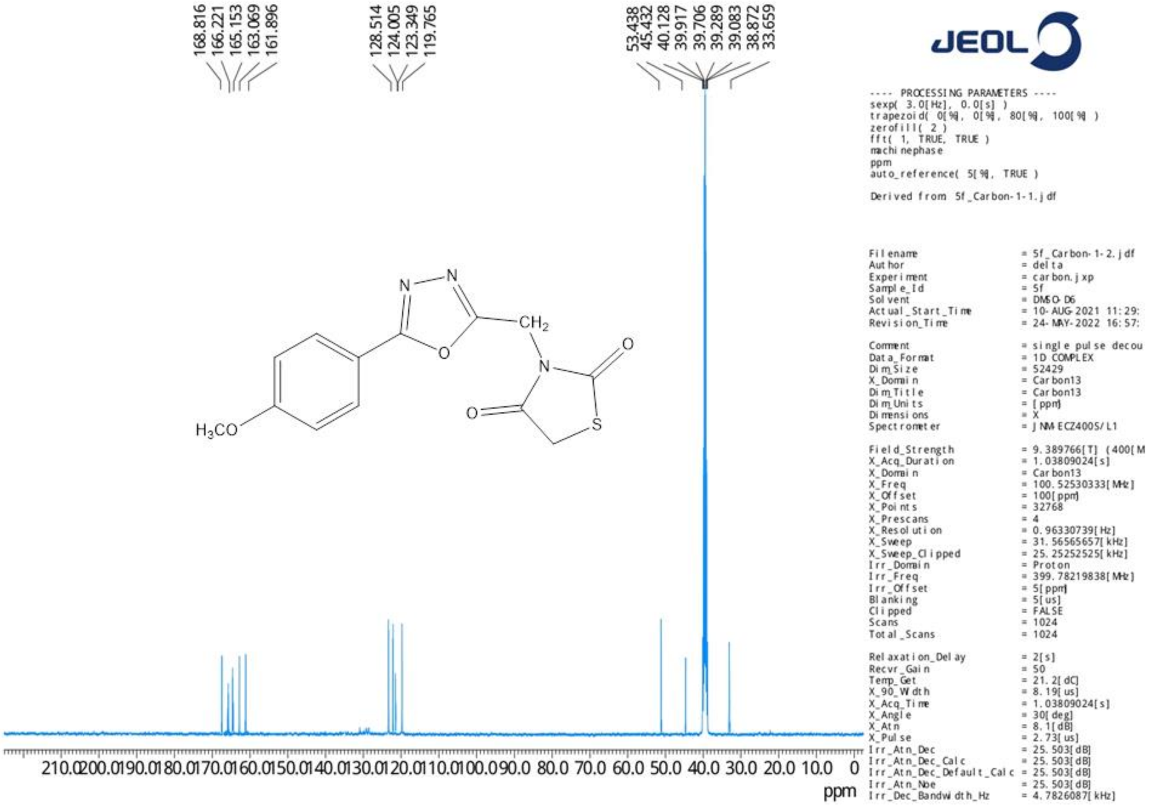

Compound 5f: 3-((5-(4-methoxyphenyl)-1,3,4-oxadiazol-2-yl)methyl)thiazolidine-2,4-dione

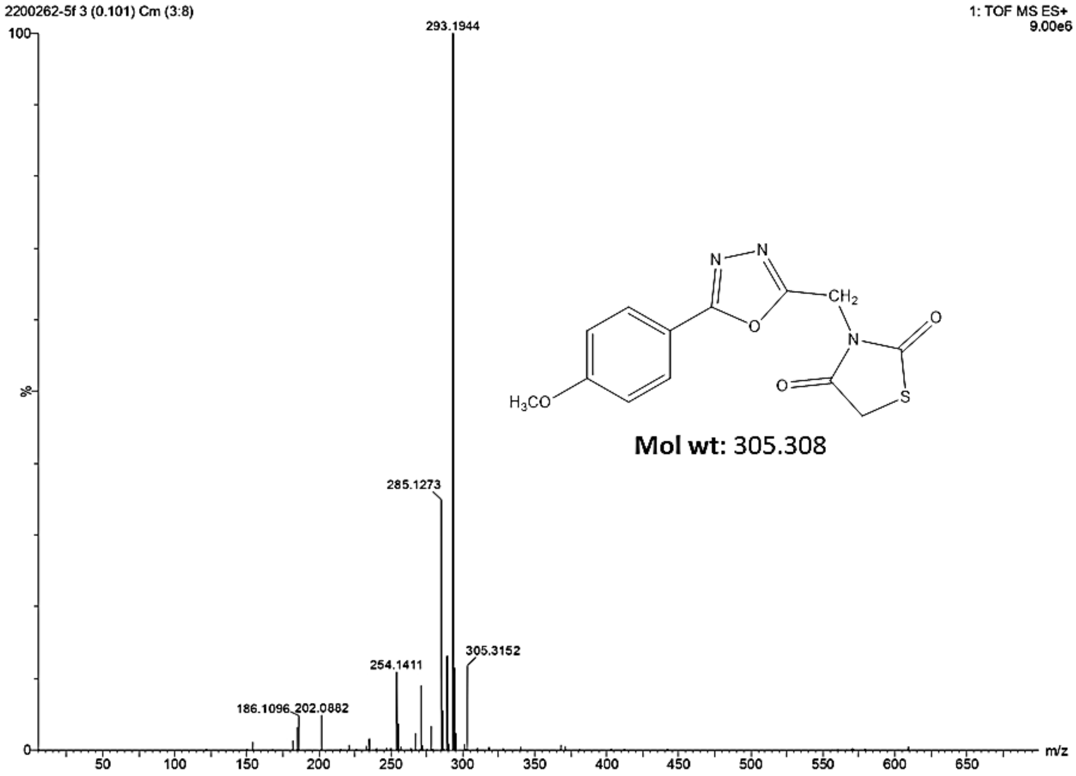

Compound 5g: 3-((5-(4-chlorophenyl)-1,3,4-oxadiazol-2-yl)methyl)thiazolidine-2,4-dione

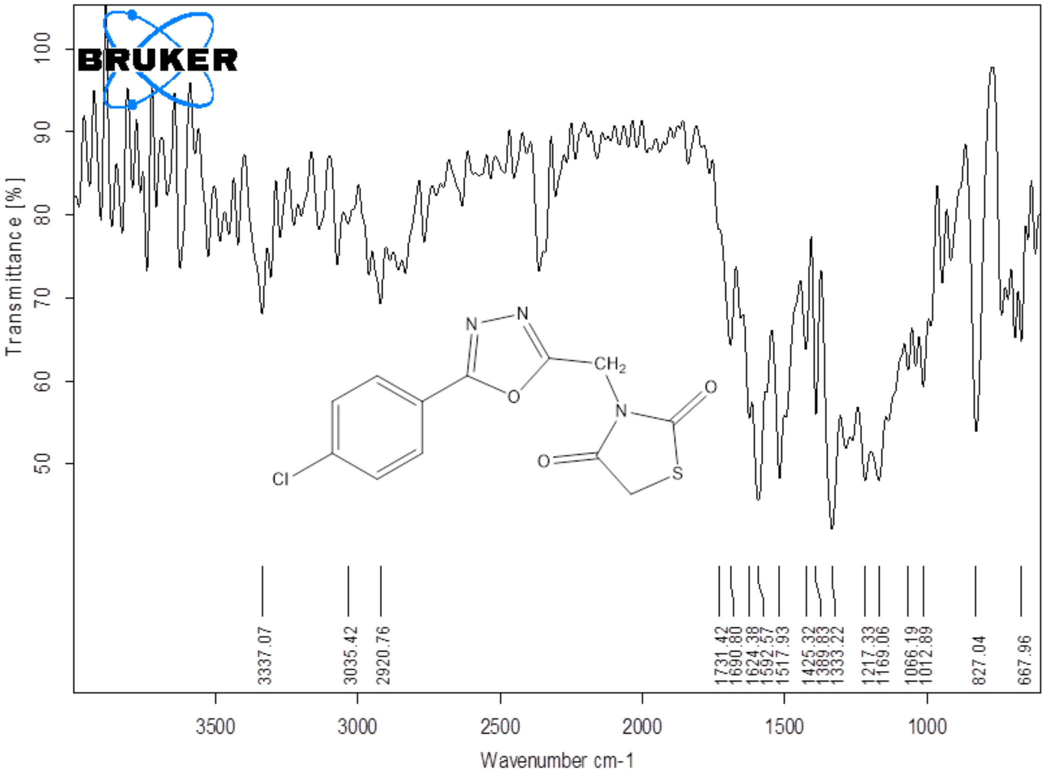

Compound 5g: 3-((5-(4-chlorophenyl)-1,3,4-oxadiazol-2-yl)methyl)thiazolidine-2,4-dione

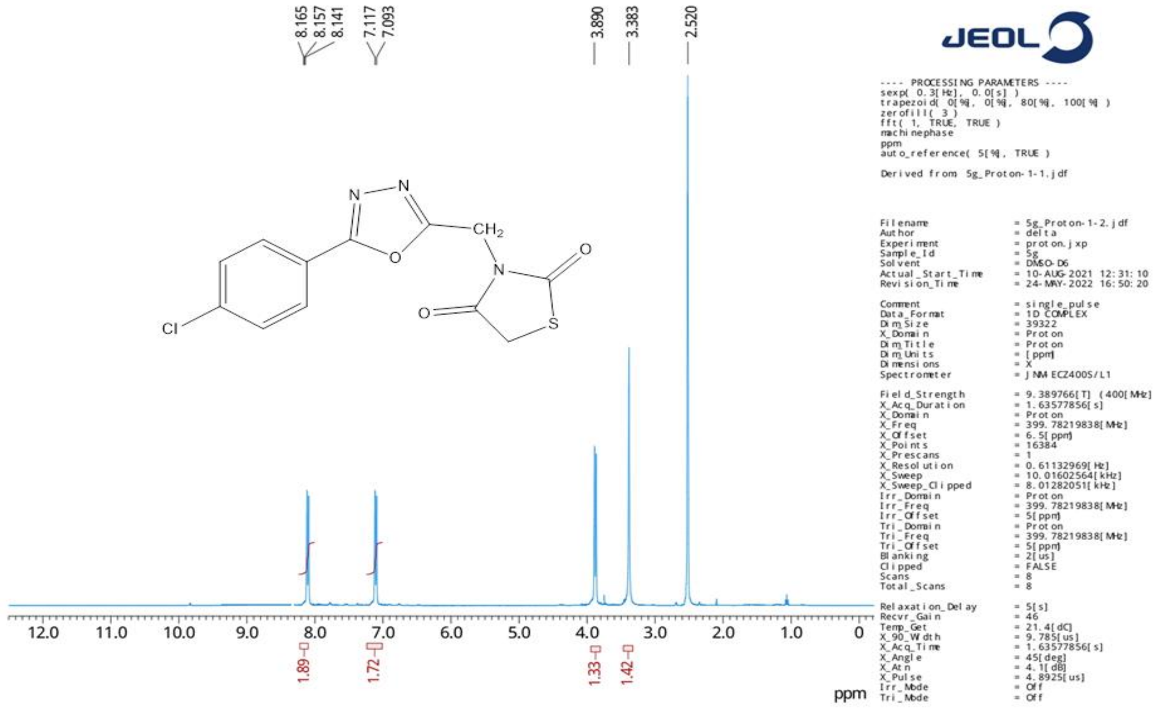

Compound 5g: 3-((5-(4-chlorophenyl)-1,3,4-oxadiazol-2-yl)methyl)thiazolidine-2,4-dione

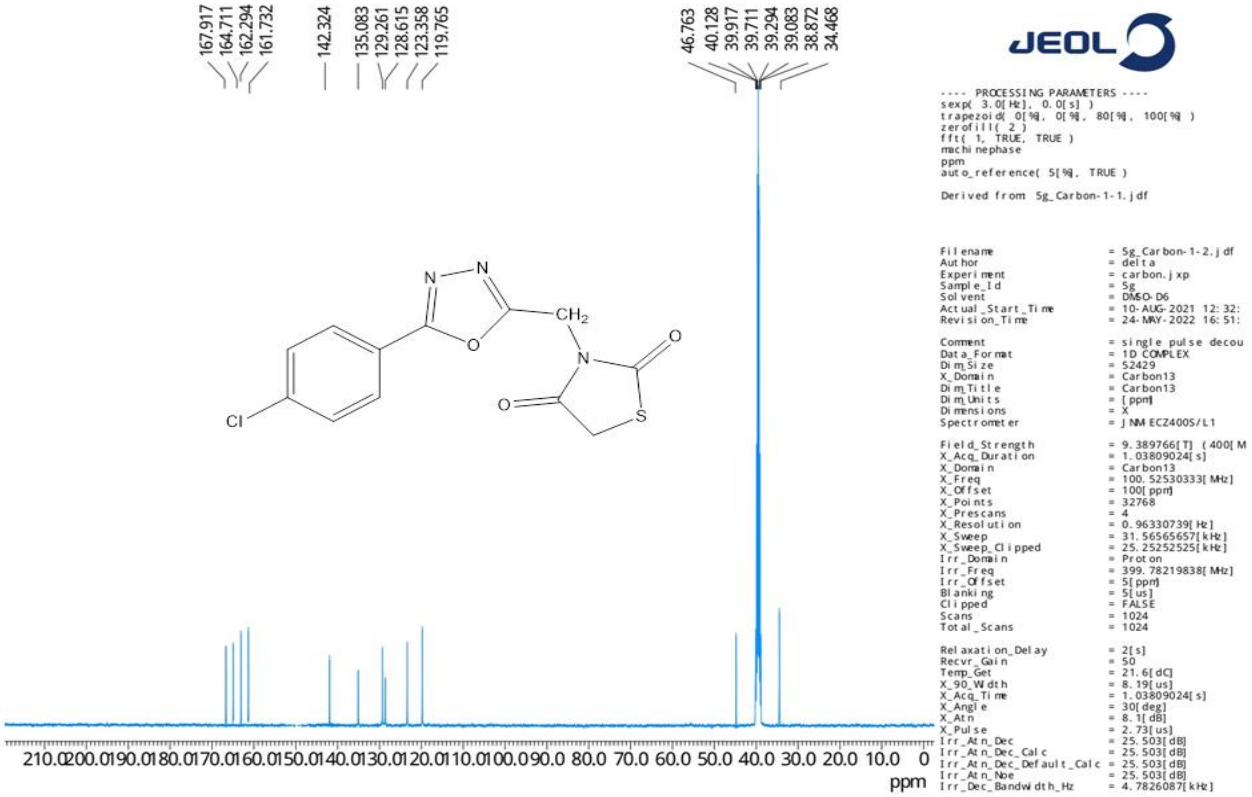

Compound 5g: 3-((5-(4-chlorophenyl)-1,3,4-oxadiazol-2-yl)methyl)thiazolidine-2,4-dione

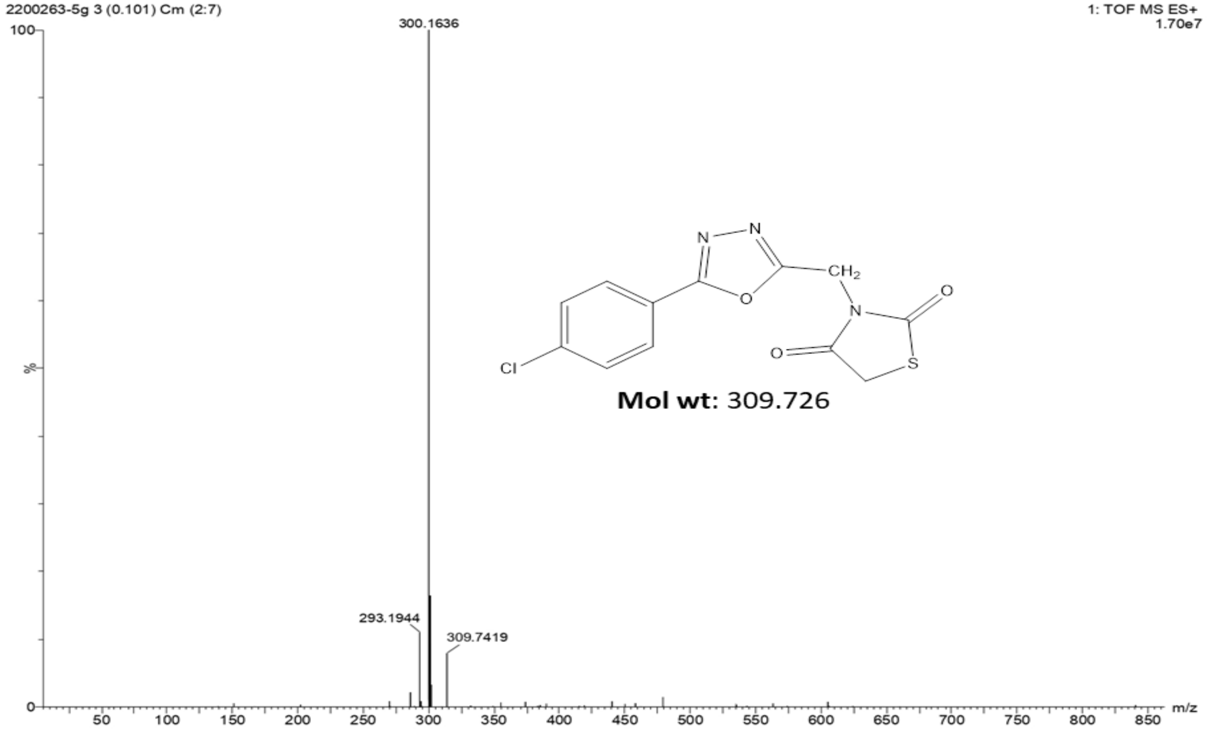

Compound 5h: 3-((5-(2-chloro-4-nitrophenyl)-1,3,4-oxadiazol-2-yl)methyl)thiazolidine-2,4-dione

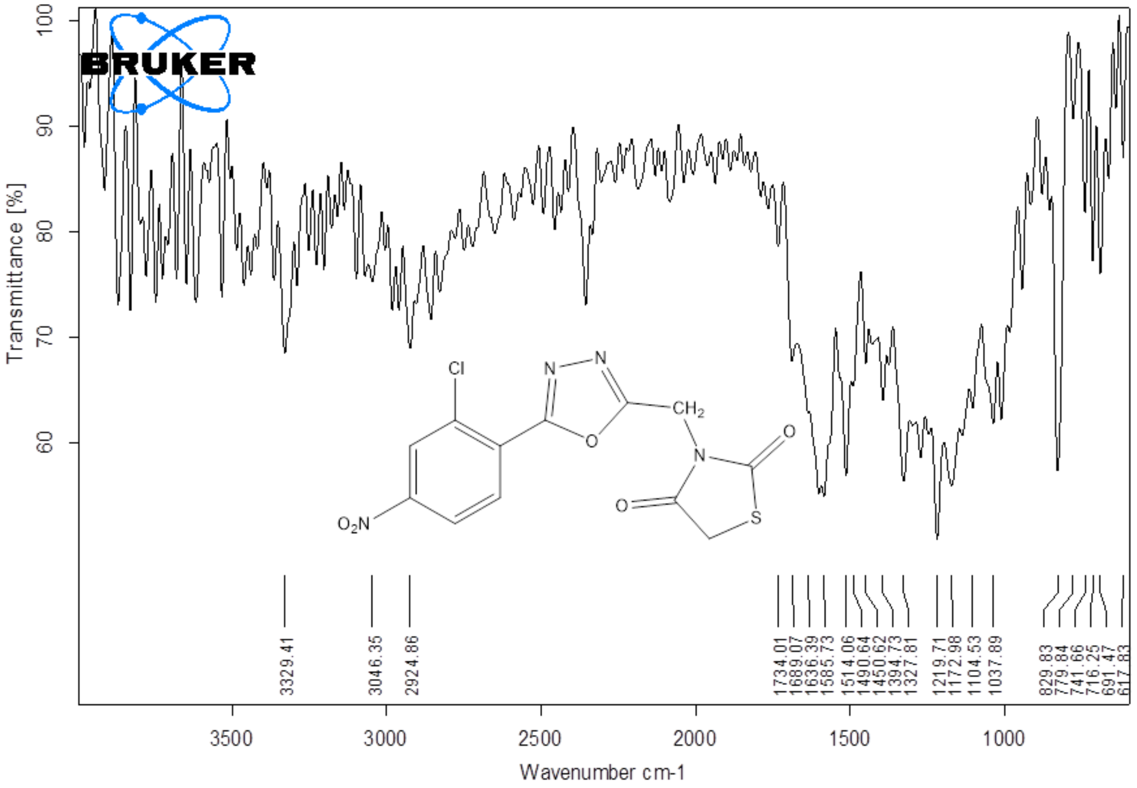

Compound 5h: 3-((5-(2-chloro-4-nitrophenyl)-1,3,4-oxadiazol-2-yl)methyl)thiazolidine-2,4-dione

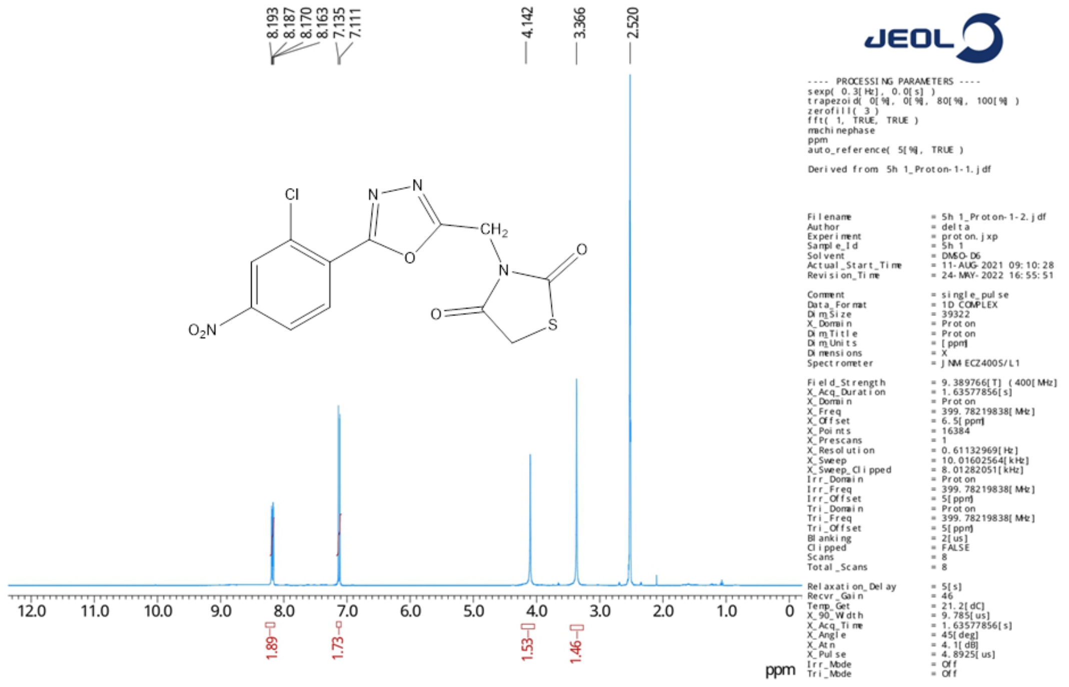

Compound 5h: 3-((5-(2-chloro-4-nitrophenyl)-1,3,4-oxadiazol-2-yl)methyl)thiazolidine-2,4-dione

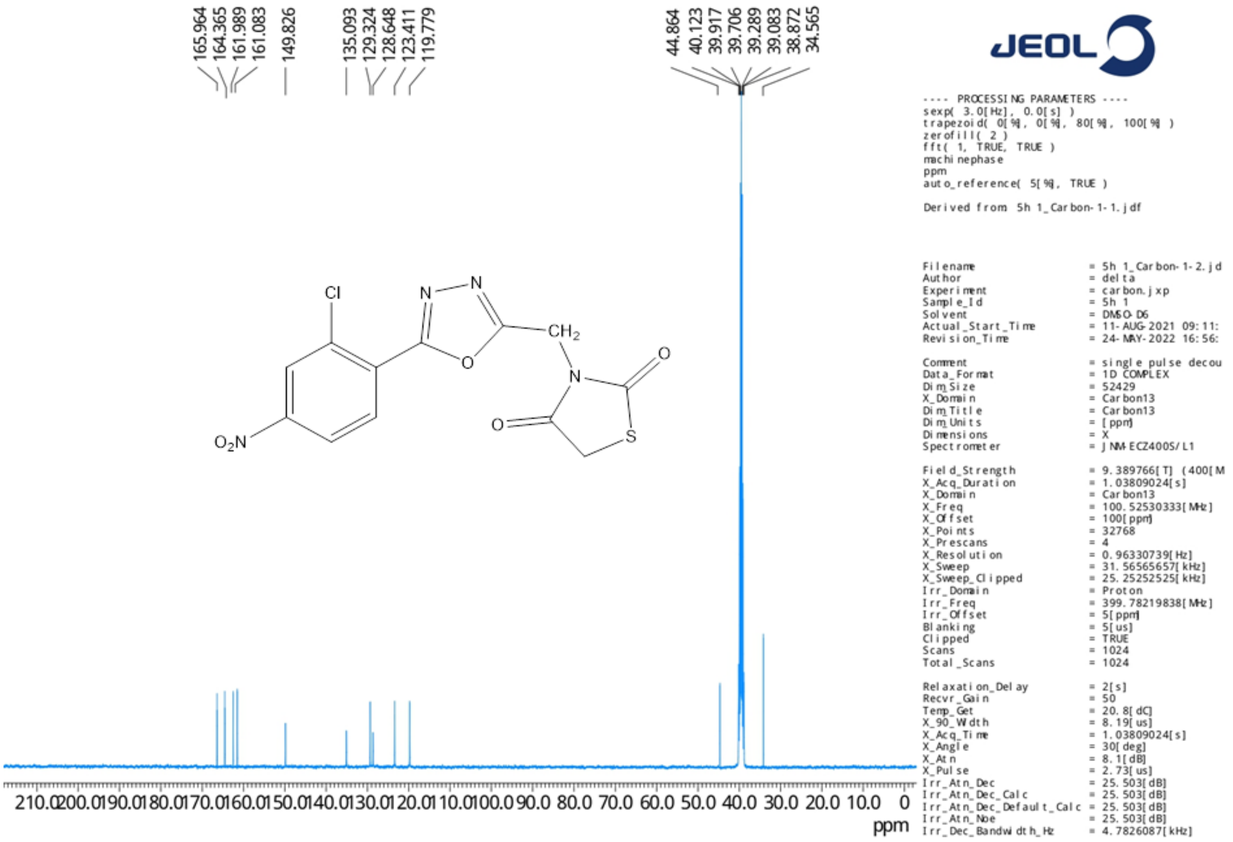

Compound 5h: 3-((5-(2-chloro-4-nitrophenyl)-1,3,4-oxadiazol-2-yl)methyl)thiazolidine-2,4-dione

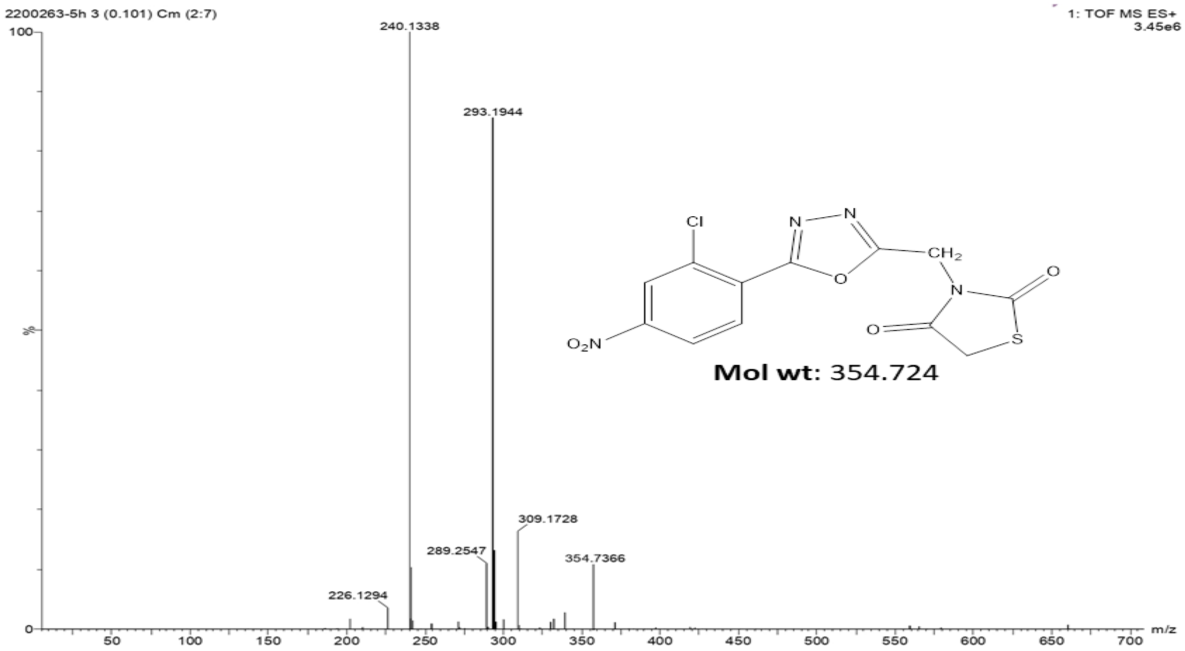

Compound 5i: 3-((5-(4-nitrophenyl)-1,3,4-oxadiazol-2-yl)methyl)thiazolidine-2,4-dione

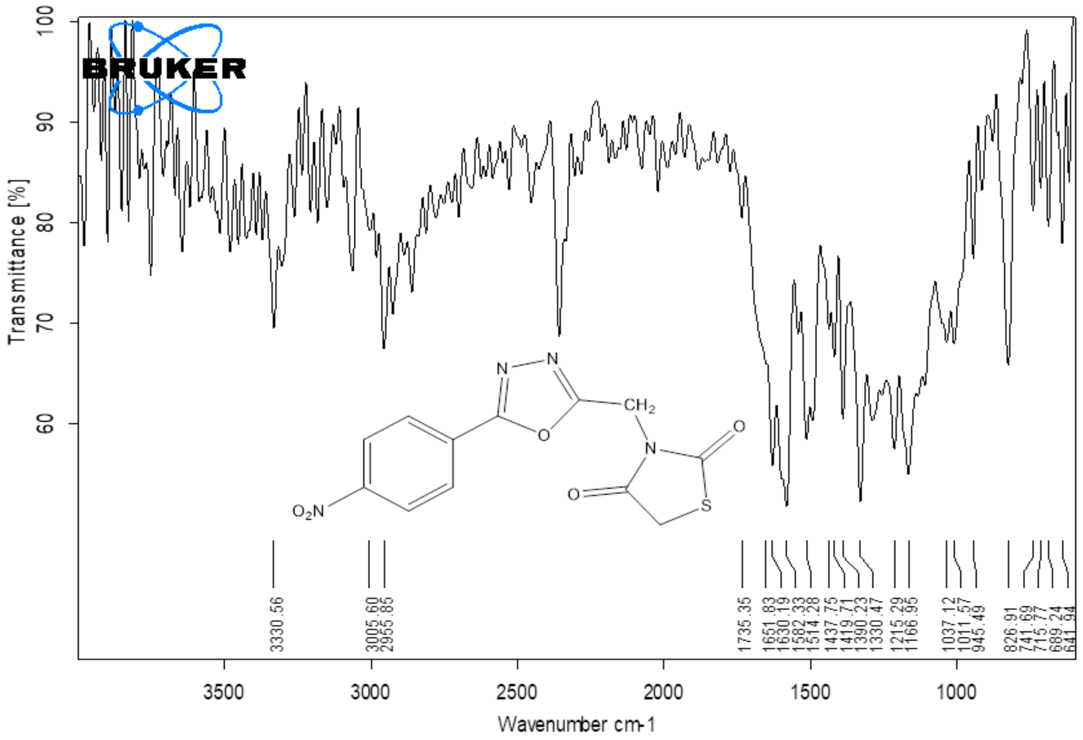

Compound 5i: 3-((5-(4-nitrophenyl)-1,3,4-oxadiazol-2-yl)methyl)thiazolidine-2,4-dione

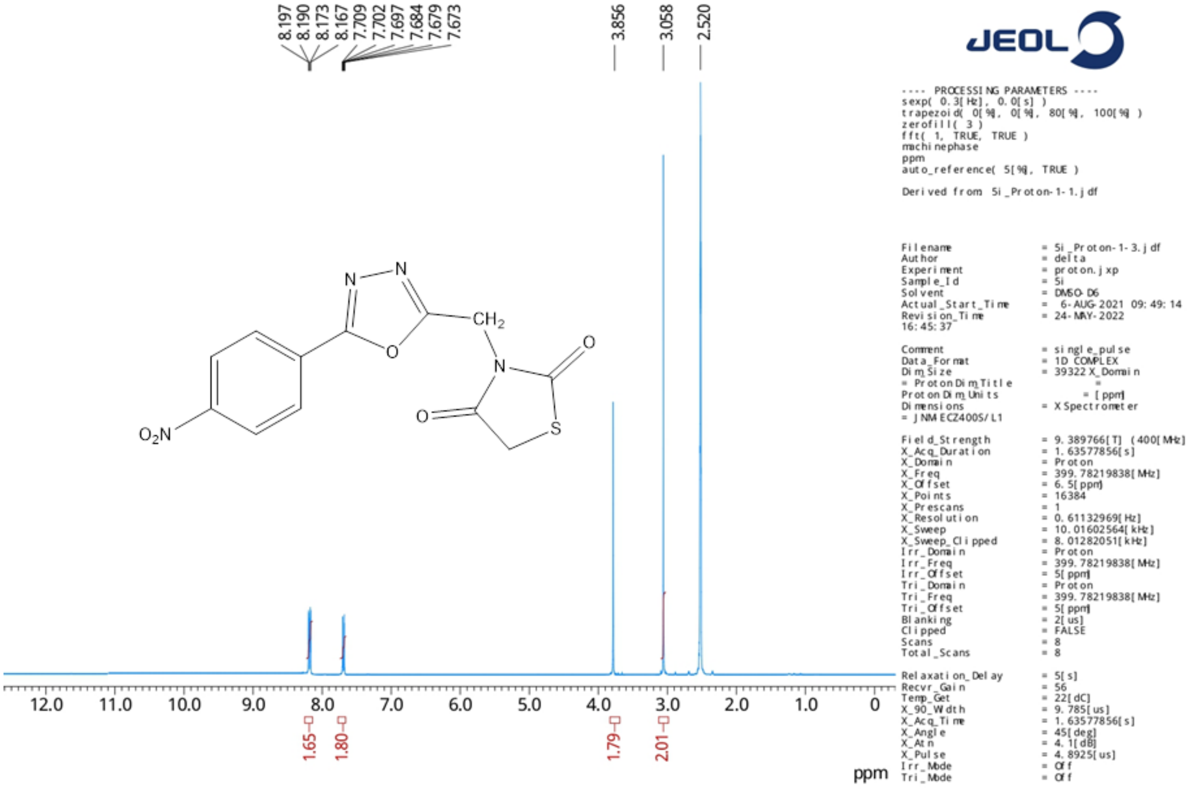

Compound 5i: 3-((5-(4-nitrophenyl)-1,3,4-oxadiazol-2-yl)methyl)thiazolidine-2,4-dione

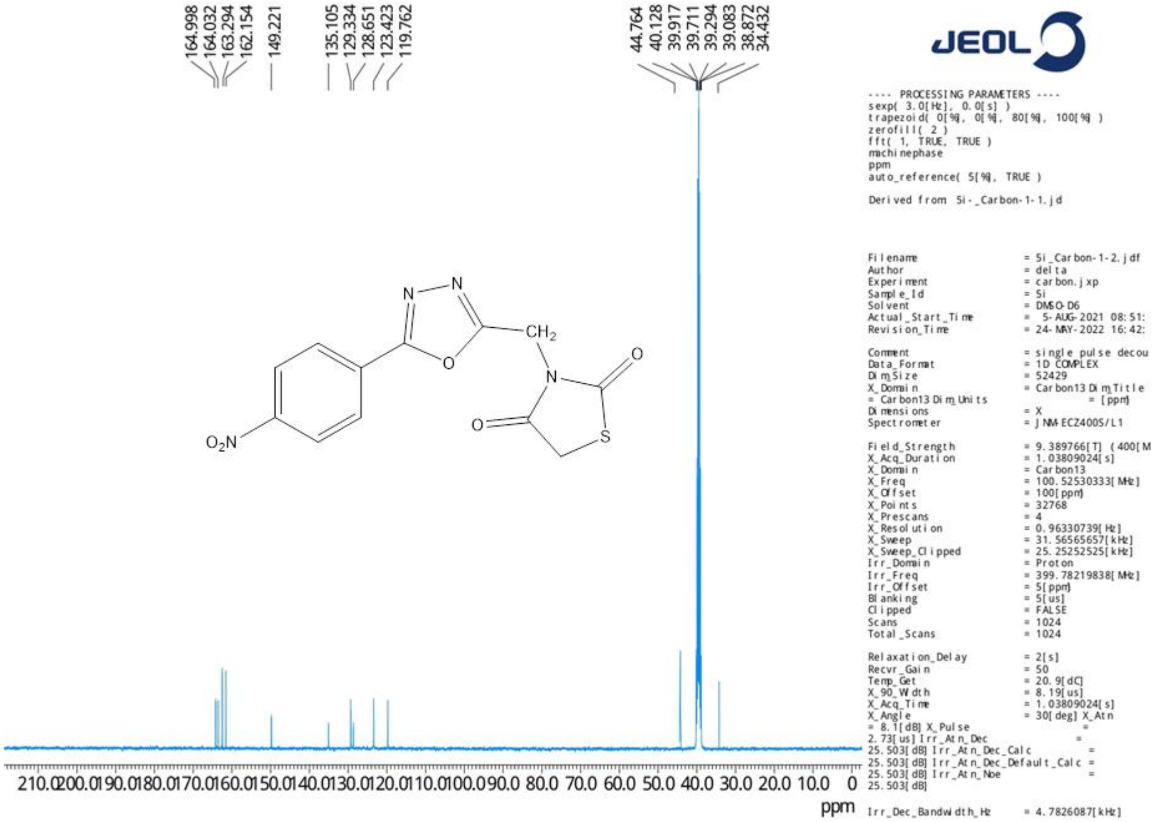

Compound 5i: 3-((5-(4-nitrophenyl)-1,3,4-oxadiazol-2-yl)methyl)thiazolidine-2,4-dione

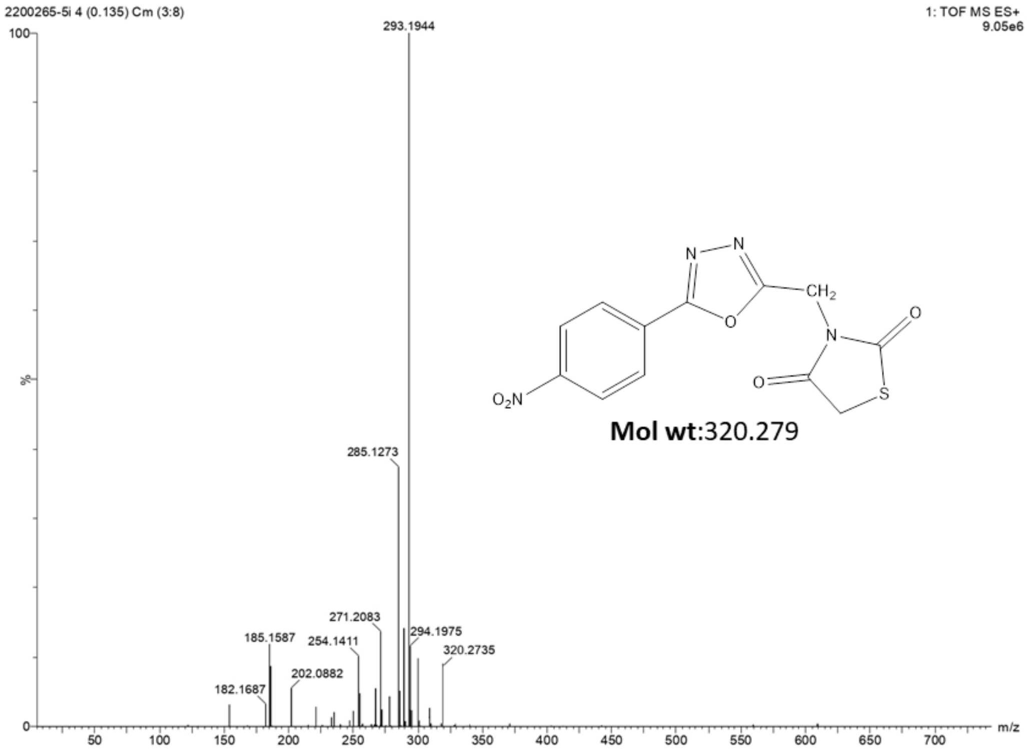

Compound 5j: 3-((5-(2,4-dihydroxyphenyl)-1,3,4-oxadiazol-2-yl)methyl)thiazolidine-2,4-dione

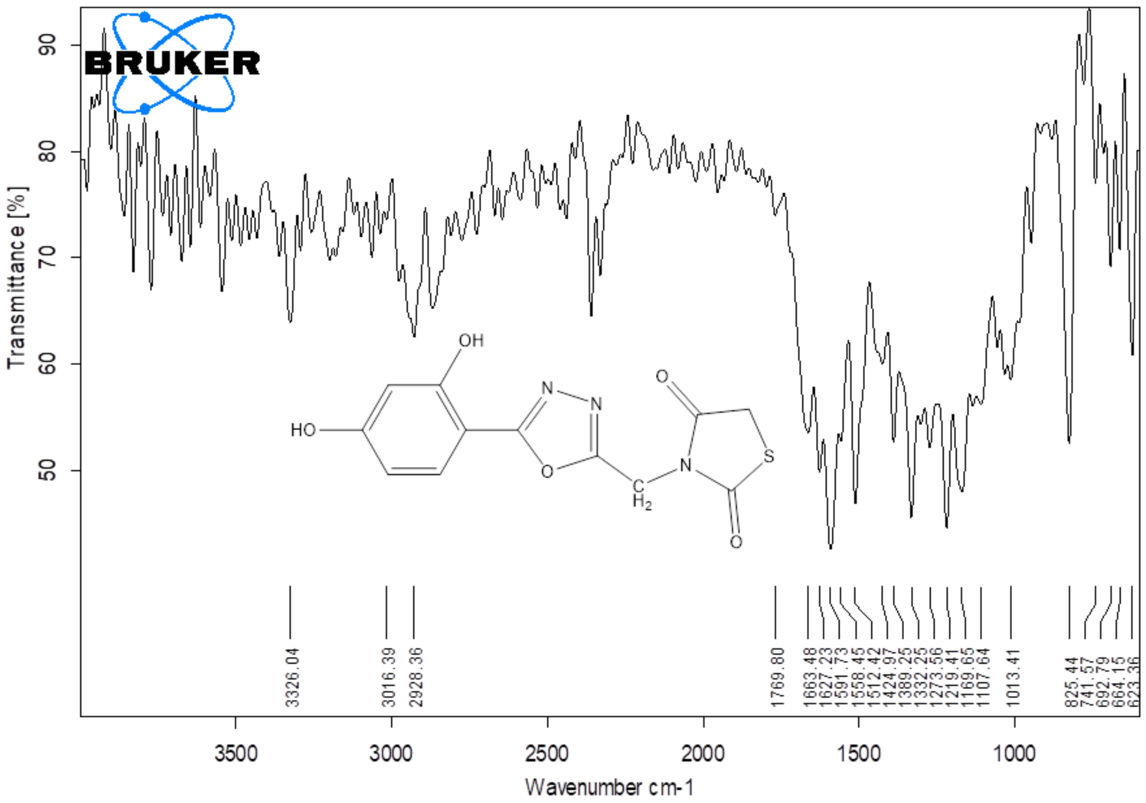

Compound 5j: 3-((5-(2,4-dihydroxyphenyl)-1,3,4-oxadiazol-2-yl)methyl)thiazolidine-2,4-dione

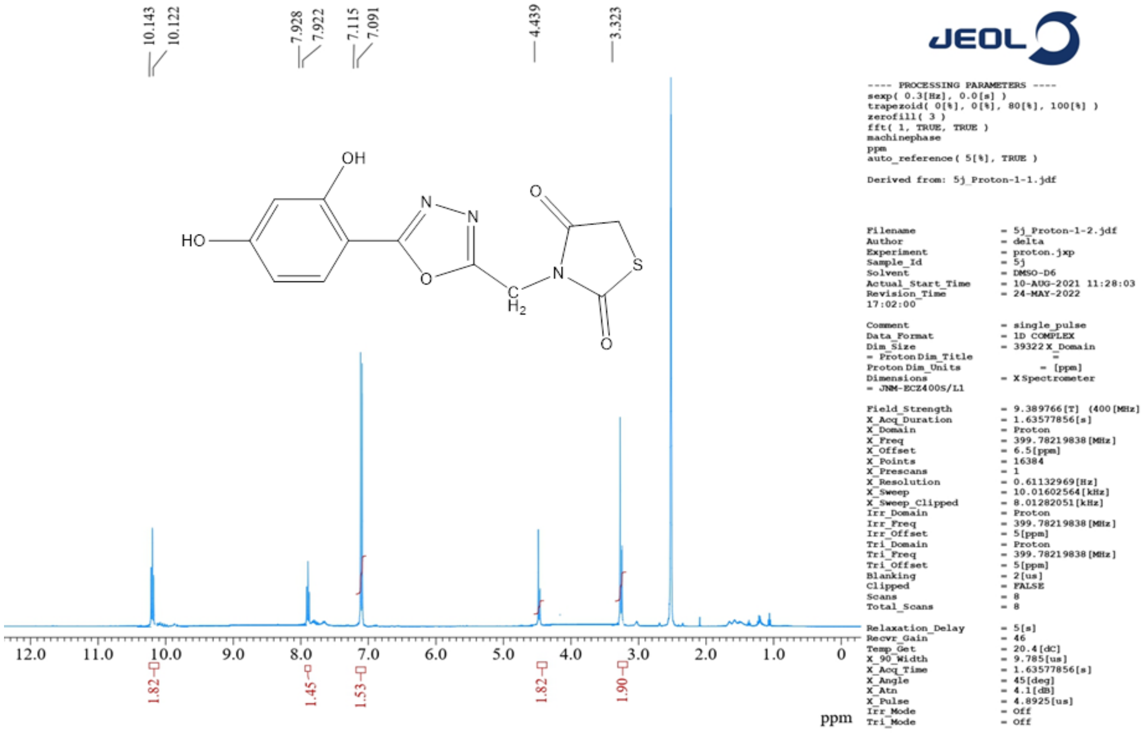

Compound 5j: 3-((5-(2,4-dihydroxyphenyl)-1,3,4-oxadiazol-2-yl)methyl)thiazolidine-2,4-dione

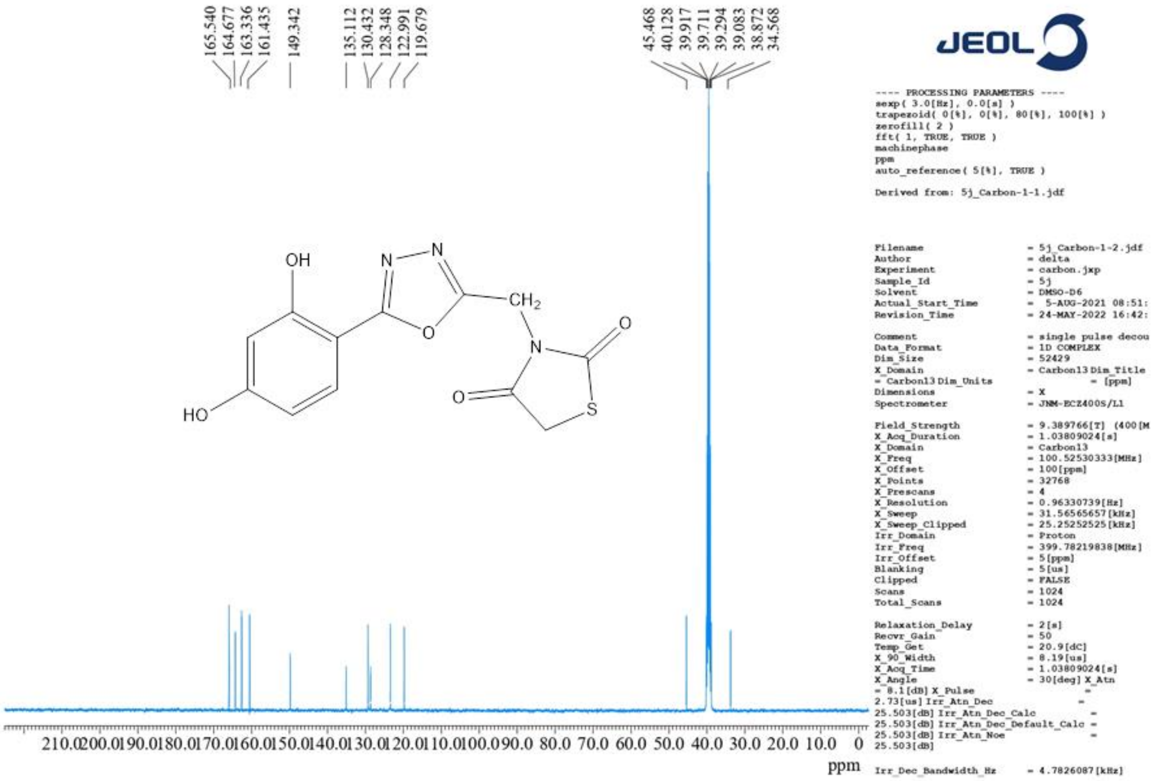

Compound 5j: 3-((5-(2,4-dihydroxyphenyl)-1,3,4-oxadiazol-2-yl)methyl)thiazolidine-2,4-dione

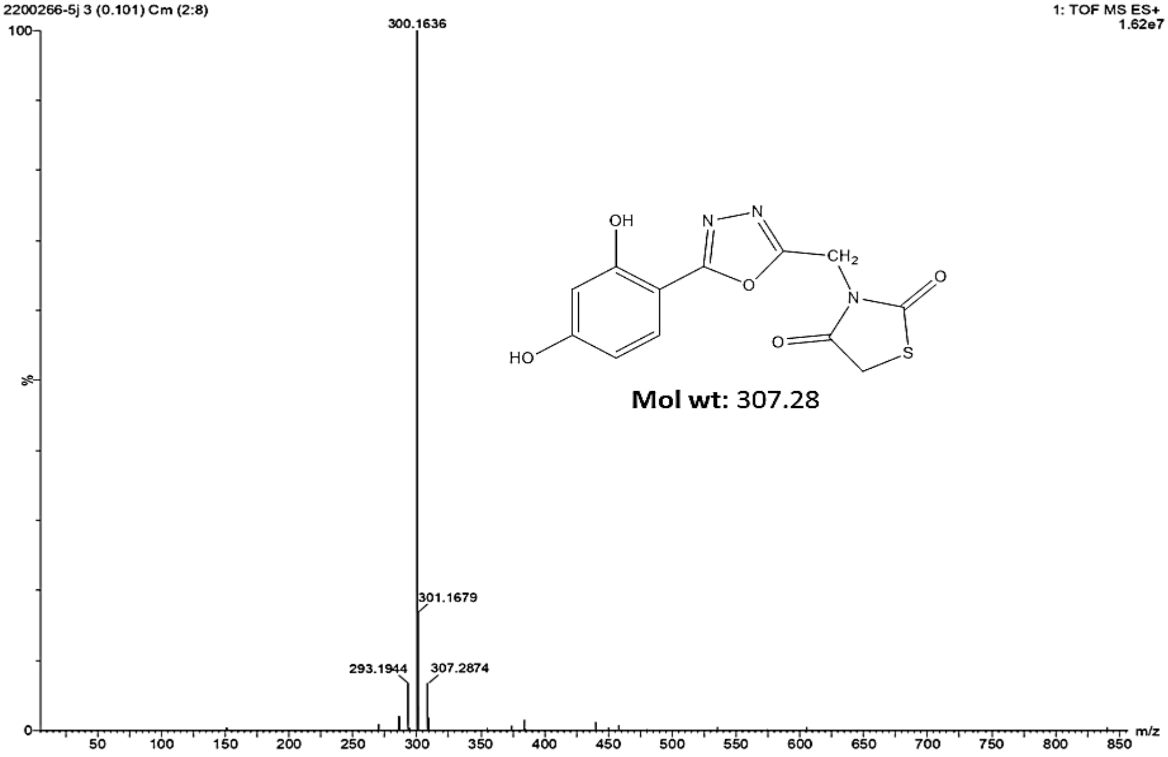

Supplement: RA-013-D2RA07247E-s001 [file RA-013-D2RA07247E-s001.pdf]
